# Predictors of Response to Autologous Dendritic Cell Therapy in Glioblastoma Multiforme

**DOI:** 10.3389/fimmu.2018.00727

**Published:** 2018-05-29

**Authors:** Chia-Ing Jan, Wan-Chen Tsai, Horng-Jyh Harn, Woei-Cherng Shyu, Ming-Chao Liu, Hsin-Man Lu, Shao-Chih Chiu, Der-Yang Cho

**Affiliations:** ^1^Division of Molecular Pathology, Department of Pathology, China Medical University and Hospital, Taichung, Taiwan; ^2^Department of Pathology, China Medical University and Beigang Hospital, Yunlin, Taiwan; ^3^Department of Medicine, China Medical University, Taichung, Taiwan; ^4^Center for Cell Therapy, China Medical University Hospital, Taichung, Taiwan; ^5^The Buddhist Tzu Chi Bioinnovation Center, Buddhist Tzu Chi University, Haualien, Taiwan; ^6^Department of Pathology, Buddhist Tzu Chi General Hospital and Buddhist Tzu Chi University Haualien, Haualien, Taiwan; ^7^Translational Medicine Research Center, China Medical University Hospital, Taichung, Taiwan; ^8^Center for Neuropsychiatry, Department of Neurology, China Medical University Hospital, Taichung, Taiwan; ^9^Institute of Clinical Medical Science, China Medical University, Taichung, Taiwan; ^10^Department of Psychology, Asia University, Taichung, Taiwan; ^11^Graduate Institute of Biomedical Sciences, China Medical University, Taichung, Taiwan; ^12^Graduate Institute of Immunology China Medical University, Taichung, Taiwan; ^13^Department of Neurosurgery, Neuropsychiatric Center, China Medical University Hospital, Taichung, Taiwan

**Keywords:** autologous dendritic cell/tumor antigen, glioblastoma multiforme, tumor-infiltrating lymphocytes, immune checkpoints, peripheral blood mononuclear cell, programmed death protein 1 (PD-1^+^), cytotoxic T-lymphocytes (CD8^+^), PD-1^+^/CD8^+^ ratio

## Abstract

**Background:**

Glioblastoma (GBM) is the most common and lethal primary malignant glioma in adults. Dendritic cell (DC) vaccines have demonstrated promising results in GBM clinical trials. However, some patients do not respond well to DC therapy, with survival rates similar to those of conventional therapy. We retrospectively analyzed clinical and laboratory data to evaluate the factors affecting vaccine treatment.

**Methods:**

Forty-seven patients with *de novo* GBM were enrolled at China Medical University Hospital between 2005 and 2010 and divided into two subgroups. One subgroup of 27 patients received postsurgical adjuvant immunotherapy with autologous dendritic cell/tumor antigen vaccine (ADCTA) in conjunction with conventional treatment of concomitant chemoradiotherapy (CCRT) with temozolomide. The other 20 patients received only postsurgical conventional treatment without immunotherapy. Immunohistochemistry for CD45, CD4, CD8, programed death ligand 1 (PD-L1), and programed death 1 (PD-1) was performed on sections of surgical tumor specimens and peripheral blood mononuclear cells (PBMCs). Pearson’s correlation, Cox proportional hazard model, and Kaplan–Meier analyses were performed to examine the correlations between the prognostic factors and survival rates.

**Results:**

Younger age (<57 years), gross total resection, and CCRT and PD-1^+^ lymphocyte counts were significant prognostic factors of overall survival (OS) and progression-free survival (PFS) in the ADCTA group. Sex, CD45^+^ lymphocyte count, CD4^+^ or CD8^+^ lymphocyte count, tumor PD-L1 expression, isocitrate dehydrogenase 1 mutation, and O6 methylguanine-DNA methyltransferase promoter methylation status were not significant factors in both groups. In the ADCTA group, patients with tumor-infiltrating lymphocytes (TILs) with a lower PD-1^+^/CD8^+^ ratio (≤0.21) had longer OS and PFS (median OS 60.97 months, *P* < 0.001 and PFS 11.2 months, *P* < 0.008) compared to those with higher PD-1^+^/CD8^+^ ratio (>0.21) (median OS 20.07 months, *P* < 0.001 and PFS 4.43 months, *P* < 0.008). Similar results were observed in patients’ PBMCs; lymphocyte counts with lower PD-1^+^/CD8^+^ ratio (≤0.197) had longer OS and PFS. There was a significant correlation of PD-1^+^/CD8^+^ ratio between TILs and PBMCs (Pearson’s correlation *R*^2^ = 0.6002, *P* < 0.001). By contrast, CD4^−^, CD8^−^, but PD-1^+^, CD45^+^ tumor-infiltrating lymphocytes have no impact on OS and PFS (*P* = 0.073 and *P* = 0.249, respectively).

**Conclusion:**

For patients receiving DC vaccine adjuvant therapy, better outcomes are predicted in patients with younger age, with TILs or PBMCs with lower PD-1^+^/CD8^+^ ratio, with gross tumor resection, and receiving CCRT.

## Introduction

Glioma is a commonly occurring form of brain tumor, and high-grade gliomas are the most common malignant tumors of the central nervous system (1, 2). Glioblastoma multiforme (GBM) is the most lethal, with a mortality rate of 88% within 3 years (3). With standard treatments, GBM prognosis remains poor, with a median overall survival (OS) of 14.6 months for newly diagnosed GBM treated with temozolomide (TMZ) and a median OS of 7.4 months for recurrent GBM (4, 5).

Even with advances in surgical procedures, radio-therapeutic technologies, and discovery of new chemotherapeutic agents such as bevacizumab (Avastin) (6, 7), patients with GBM still have a dismal prognosis, which is approximately 19 months for median OS (8), although some reports showed 2-year survival rates of 40–50% (9, 10) to date. Survival rates of patients with glioblastoma are relatively low, and the failure of treatments is mostly due to recurrence. GBM has a low survival rate attributed to unique treatment limitations such as a high cell proliferation, invasive infiltration, tumor location, and poor understanding of the tumor pathophysiology (11).

In recent years, cancer immunotherapy has been pursued by exploitation of dendritic cells (DCs), which are “professional” antigen processing and presenting cells utilized to induce specific antitumor responses (12). DCs are very potent antigen-presenting cells that play a key role in the initiation of the immune response, and are considered a promising tool for immunotherapy (13, 14). DC-based therapy may provide a way for cytotoxic T lymphocytes (CTLs), natural killer cells, and cytokines to directly or indirectly kill tumor cells. Active immunotherapy using DCs induces an antigen-specific T-cell response to tumor antigens, and recent reports have shown the feasibility, safety, and bioactivity of autologous DC vaccines for GBM, even with recurrent tumors (15, 16).

Clinical trials for GBM with DC vaccine treatment may increase OS time from control group of 13.1 months to DC vaccine group of 15.7 to 35.9 months, according to different trials (12). Since 2001, various research groups have attempted to use DC-based immunotherapy (also called DC vaccines in some studies) in treatment of malignant gliomas (14, 17–19), and have reported induction of glioma-specific antitumor immune responses and apparent survival benefits for some patients. The median OS in trials recruiting patients with newly diagnosed GBM varied between 16.0 and 38.4 months, whereas for recurrent GBM, it ranged between 9.6 and 35.9 months (20–22).

To evaluate the efficacy of DC treatment, two investigator-initiated trials have been conducted in Taiwan, and the results have been published as two peer-review journal articles (23, 24). In Cho’s series, the 2-year survival rate was 47.2%, and the 3-year survival rate was 26.6%.

Similar to other studies, the results of our early experience have revealed a significant benefit for patients with high-grade glioblastoma; however, survival in DC vaccine-treated GBM patients varied (25). Recently, studies of immune checkpoints have provided many dramatic breakthroughs in tumor immune therapy, and checkpoint blockade has shown effectiveness in lung and breast tumors or others (26–33). The tumor microenvironment is complex. Immune checkpoint inhibitors such as programed death 1 protein (PD-1) and programmed death ligand 1 (PD-L1) tremendously influence the immune therapeutic outcome in many types of tumor including GBM (34–37). Many clinical trials of checkpoint blockade are under way (35, 38, 39). Studies in various cancer models have suggested that immunological checkpoint mechanisms such as the PD-1/PD-L1 pathway may contribute to self-tolerance and induce CD8^+^ T-cell exhaustion in the tumor microenvironment (28, 40–43). Recently, the concept of PD-1/PD-L1 in regulating the response to glioma with DC vaccine treatment was also evaluated and proved by preclinical evidence *in vitro* and *in vivo* in animal studies (44, 45). Therefore, we retrospectively analyzed clinical data and paraffin blocks from our previous study for improving the effectiveness of autologous DC treatment of GBM.

## Materials and Methods

### Patients

This was a retrospective review of 47 samples from patients in a previous clinical study (24) between November 2005 and April 2010 following a new diagnosis of histologically confirmed glioblastoma multiforme (GBM, WHO grade 4 astrocytoma). Patients were between the ages of 14 and 70 years at diagnosis. Inclusion criteria included a Karnofsky performance score (KPS) of at least 70 before surgery and adequate hematologic, renal, and hepatic function [hemoglobin ≥8 g/dL, platelets, ≥100,000/μL, white blood cell count >2,000/μL, absolute neutrophil count ≥1,000/μL, serum blood urea nitrogen <25 mg/dL, serum creatinine <1.8 mg/dL, creatinine clearance >50 mL/min, both serum ALT and serum AST ≤ 3 × the upper limit of normal (ULN), alkaline phosphatase (AP) ≤ 3 × ULN, serum total bilirubin < mg/dL, and prothrombin time and partial thromboplastin time ≤ 1.5 × ULN]. This study was carried out in accordance with the recommendations of ethics guidelines of the institutional hospital with written informed consent from all subjects. All subjects gave written informed consent in accordance with the Declaration of Helsinki. The ethics committee at China Medical University Hospital (Taiwan) approved the study protocol (approval no. CMUH106-REC1-098).

### Study Design

**Figure 1:**
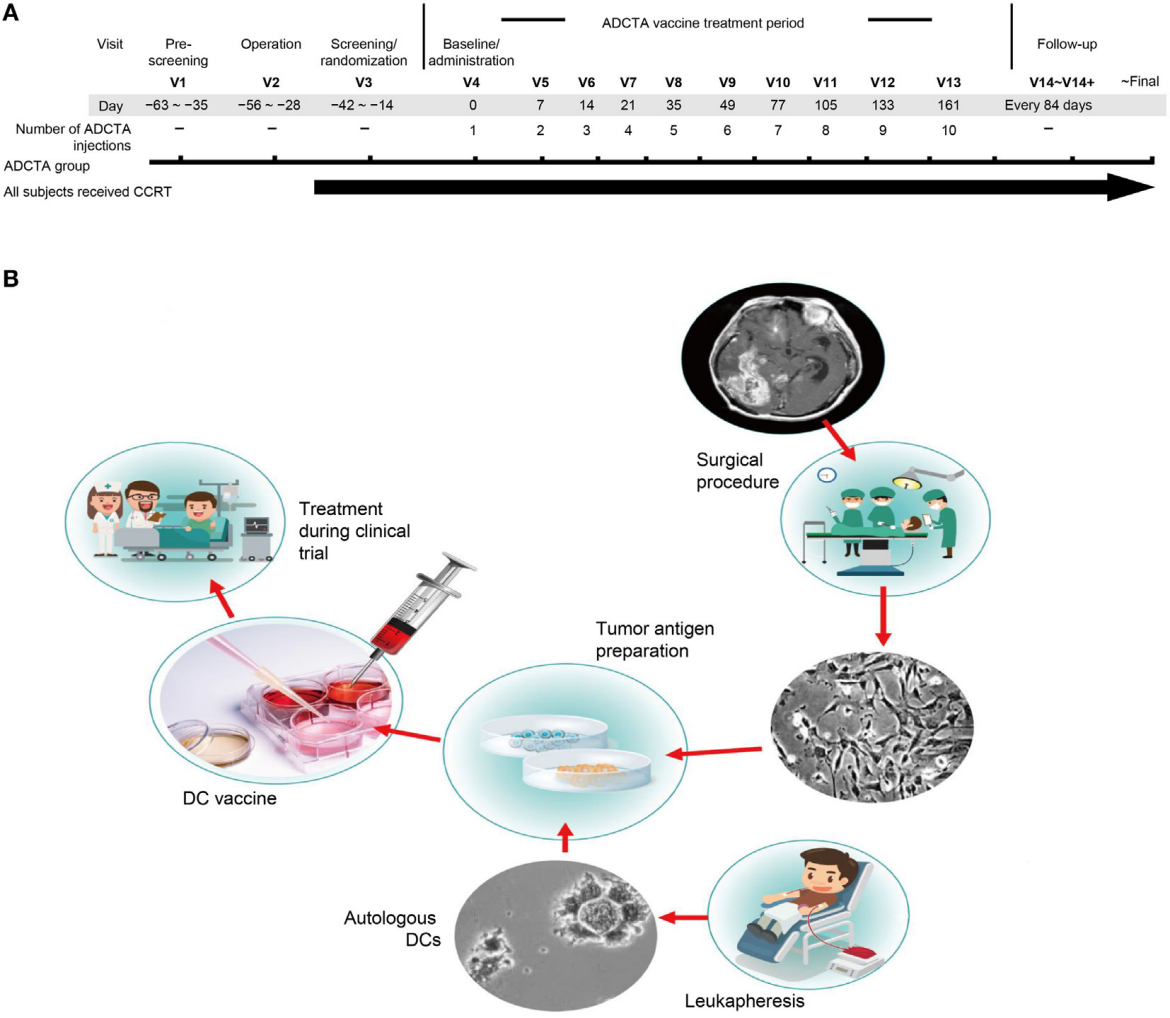
Treatment schema and vaccine preparation. Clinical schematic diagram **(A)**. Subjects with primary GBM will be consent for operation and concomitant chemoradiotherapy (CCRT). Subjects assigned to the ADCTA group will be designated to receive dendritic cell (DC) vaccination ten times following the clinical trial schedule after operation. V: visits to hospital, numbers following indicates times of visit. DC vaccine manufacturing protocol **(B)**. In China Medical University Hospital, the DC vaccine is produced in laboratories that meet the requirements of Good Tissue Practices and Good Manufacturing Practices. The final product is used in a clinical trial of autologous DC therapy for GBM patients between years 2005 and 2010.

The primary objective was to examine the initial tumor specimen or peripheral blood mononuclear cell (PBMC) expression of CD45, CD4, CD8, PD-L1, and PD-1 in GBM patients who received conventional therapy, compared with those who received conventional therapy with adjuvant autologous dendritic cell tumor antigen (ADCTA) vaccine. The conventional treatment was defined as tumor resection or biopsy (non-resectable) and subsequent concomitant chemoradiotherapy (CCRT) with TMZ, according to the guidelines suggested by Stupp et al. (46) (we defined this as the reference group). The add-on study design included an ADCTA vaccine treatment period, a posttreatment tracking period, and a retrospective pathological analysis (Figure 1A). The ADCTA vaccine therapy began 1–2 months post-surgery in conjunction with concomitant CCRT and TMZ. The vaccination protocol for this 10-injection course was four times every 2 weeks followed by monthly six times for a course of 8 months. For patients who were too weak or for other reasons unable to complete the full 10 injections, a minimum of 4 injections was required; otherwise, the patient was excluded from the study. In the reference group, patients underwent surgery followed by concomitant CCRT with TMZ only.

### Preparation of DC-Based Vaccine

In the ADCTA group, the protocol for DC-based vaccine preparation was based on 2011 and 2012 reports by Chang et al. and Cho et al. (23, 24), and the schematic diagram is represented in Figure 1B.

In brief, fresh tumor tissues removed in the operating room were collected and chopped into small pieces, and then processed by the Brain tumor dissociation kits (130-095-942; MACS^®^, Miltenyi Biotec, Germany). After cell culture, at least 100–150 million tumor cells were generated to provide sufficient tumor antigens. Cells were irradiated with 20 Gy and then lysed by quick freezing and thawing to produce tumor antigens. At least 2 mg of total protein from the cell lysates were collected from the supernatant after centrifugation for use as the source of tumor antigens. We collected the patient’s PBMC (5.0 × 10^9^ cells/mL) from peripheral blood through leukapheresis. Generally, the leukapheresis was performed in 1 month after subjects had an operation. The monocytes were enriched by 2-h attachment method on the plastic dish at 37°C. The isolated monocytes were cultured in CTS™ AIM V™ Medium (Invitrogen, Carlsbad, CA, USA) with various cytokine proteins for immature DC differentiation. After 7 days, prepared tumor antigens were added into the culture medium for manufacturing DC-based vaccine. Finally, we collected and washed the DC-based vaccines and then divided the cells among 12 tubes, which contain 2–5 × 10^7^ DCs cells. All DC vaccines were stored in the liquid nitrogen tank. Before used, the DC vaccine was thawed and the cells were washed with 4°C normal saline twice and added 1 mL of saline.

### Cytoblock Preparation

Peripheral blood mononuclear cells were isolated from patients enrolled in the ADCTA group and used for cytology analysis. Cytoblocks were prepared using a gel-embedding method described by Choi et al. (47).

### Immunohistochemistry (IHC) for CD45, CD4, CD8, PD-1, and PD-L1

Immunohistochemical analysis of CD45, CD4, CD8, PD-1, and PD-L1 was performed on 2-µm sequential sections of formalin-fixed, paraffin-embedded (FFPE) GBM tumor tissue, and on FFPE PBMC cytoblocks (CD45, CD4, CD8, and PD-1 only), where the results were scored independently by two board-certified pathologists (Chia-Ing Jan and Horng-Jyh Harn) with no prior knowledge of the patients’ clinical background.

Sequential tissue sections (2 µm) of the GBM tumor sections and cytoblocks isolated from each patient were attached to adhesive glass slides and baked at 70°C for 20 min prior to IHC staining of CD45, CD4, CD8, PD-1, and PD-L1, using the Leica BOND-MAX system (Leica Biosystems, Nussloch, Germany). Tissue sections were dewaxed three times in xylene for 1 min each and rehydrated through graded ethanol for 15 s each, followed by being washed three times with PBS for 15 s each. Antigen retrieval was performed by heating slides in Bond Epitope Retrieval Solution 2 (EDTA, pH 9.0; Leica, Newcastle, United Kingdom) at 100°C for 20 min. After cooling, slides were washed four times with PBS for 15 s each. Sections were incubated with primary monoclonal antibodies against CD45 (clone: X16/99, Leica Biosystems Richmond Inc, USA), at 1:200 dilution, CD4 (clone 1F6 Leica, Newcastle, United Kingdom) at 1:100 dilution, CD8 (clone 1A5, Leica, Newcastle, United Kingdom) at 1:200 dilution, or PD-1 (clone NAT105, Abcam, Cambridge, United Kingdom Abcam) and PD-L1 [clone EPR1161(2), Abcam, Cambridge, United Kingdom Abcam] at 1:200 dilution for 30 min at 25°C. Slides were washed four times with PBS for 20 s each.

For CD45, CD4, CD8, and PD-1, antigen detection was performed with Bond Polymer Refine Red Detection (Leica, Newcastle, United Kingdom). Post primary alkaline phosphatase (AP) was added and incubated for 30 min at room temperature. Subsequent polymer AP was added and incubated for 30 min at room temperature. Sections were then incubated with Leica Red Part substrate for 15 min at room temperature. Finally, slides were counterstained with methyl green (Dako, Glostrup, Denmark) for 5 min and washed with PBS. Isotype controls were performed for each antibody on each specimen.

For PD-L1, Novolink Polymer Detection System (Leica, Newcastle, United Kingdom) was applied after primary antibody incubation. In brief, washed slides were incubated with Novolink Polymer for 30 min, and peroxidase activity was developed with diaminobenzidine (DAB) working solution for 5 min. Finally, the slides were counterstained with hematoxylin. An isotype control was also performed for PD-L1 on each specimen.

In GBM tissue sections stained for PD-L1, tumor cells displayed both patchy/diffuse fibrillary and geographic membraneous staining. We scored PD-L1 expression referring to the methods provided by Berghoff et al. (48) and Nduom et al. (49). When PD-L1 staining was identified with intermediate to strong staining intensity (whether fibrillary or membraneous staining pattern) ≥5% of GBM cells, we defined it as PD-L1 expression, otherwise it was defined as non-expression.

For CD45, CD4, CD8, and PD-1, 25 different high-power fields (HPFs, 400× magnification) containing the most abundant tumor infiltrating lymphocytes (TILs) and 25 random HPFs for PBMCs were counted and summed by two board certified neuropathologists (Chia-Ing Jan and Horng-Jyh Harn) who had been blinded to the patients’ treatment group and clinical history. The count for each patient’s TILs and PBMCs was determined by the two pathologists, and the level of agreement between the pathologists for the manual counting of TILs and PBMCs was compared using kappa statistics.

### Immunofluorescence (IF)

Slides were pretreated as described above for IHC. After blocking with 10% fetal bovine serum (GeneDirex, Anaheim, CA, USA) for 1 h, sections were incubated with a rabbit monoclonal antibody against human CD8 (clone SP16 Thermo Fisher, Waltham, MA, USA) for 45 min at 25°C temperature. Tissue sections were washed three times with TBS-Tween 20 at 60 rpm on a shaker for 5 min each and then incubated with Alexa Fluor 488-conjugated goat anti-rabbit secondary antibody (Thermo Fisher, Waltham, MA, USA) for 45 min at 25°C followed by three PBS-Tween 20 washes at 60 rpm on a shaker for 5 min each. PD-1 staining was performed similarly except that a mouse monoclonal mouse antibody against human PD-1 (clone NAT105, Abcam, Cambridge, United Kingdom) at 1:100 dilution and an Alexa Fluor 647-conjugated goat anti-mouse secondary antibody (Thermo Fisher, Waltham, MA, USA) at 1:500 dilution were used. Finally, sections were processed using ProLong Gold Antifade Mountant with DAPI (Thermo Fisher, Waltham, MA, USA) for DAPI nuclear stain and mounting. IF images were captured using a Leica TCS SP8 X white light laser confocal microscope.

### Isocitrate Dehydrogenase 1 (IDH1) Mutational Analysis by IHC and DNA Sequencing

For IDH1 IHC, the staining procedures were similar to those for PD-L1; the primary monoclonal antibody IDH1-R132H (clone H09, Dianova, Hamburg, Germany) was diluted at 1:100. Isotype control was also performed. Percentage of positive-stained tumor cells (cytosol + nuclear stain) and staining intensity were scored by the two pathologists. The staining intensity and percentage of positive-stained cells were then semi-quantitated into a three-tiered system (diffuse positive, focal positive, and negative) according to previous literature (50–52). For DNA sequencing, DNA was extracted from FFPE tumor specimens by scraping of tumor sections of three 5-μm-thick unstained paraffin slides. First, excess paraffin and unwanted tissue was removed via trimming with a sterile blade. Then, the remaining tissue on the slides was scraped off with a new sterile blade to remove the tissue section from the slide and transfer the tissue and DNA extraction solution using the DNeasy Blood & Tissue Kit (Qiagen, Hilden, Germany). Mutations in exon 4 of IDH1 were determined by direct sequencing in all cases. Forward and reverse primers used included 5′CGGTCTTCAGAGAAGCCATT3′ and 5′GCAAAATCACATTATTGCCAAC3′, respectively. PCR amplification was performed in a 10-mL reaction mixture containing 50 ng of tumor DNA, 2.5 µL of 10 × Dream Taq buffer, 2.5 µL of 2 mM dNTPs, each forward and reverse primer at 10 µM, and 1.25 µL of Dream Taq DNA Polymerase (Agilent Technologies, Inc., Santa Clara, CA, USA). The initial denaturation was performed at 95°C for 10 min. This was followed by 37 cycles of amplification consisting of denaturation at 95°C for 30 s, annealing at 56°C for 30 s, and extension at 72°C for 30 s.

### O6 Methylguanine-DNA Methyltransferase (MGMT) Promoter Methylation by Methylation-Specific PCR (MSP)

The O6 MGMT promoter methylation status of the GBM tumor was determined using MSP analysis. The DNA extraction method was the same as that for IDH1. The DNA extracted from tumor tissues was treated with sodium bisulfite using the EpiTect Fast DNA Bisulfite Kit (Qiagen GmbH, Hilden, Germany). Primer sequences utilized for the methylated forward and reverse primers included 5′GTTTTTAGAACGTTTTGCGTTTCGAC3′ and 5′CACCGTCCCGAA AAAAAACTCCG3′, respectively, and the unmethylated forward and reverse primers used included 5′TGTGTTTTTAGAATGTTTTGTGTTTTGAT3′ and 5′CTACCACCATCCCAAAAAAAAACT CCA3′, respectively. DNA extracted from the colon cancer SW48 cell line (obtained from American Type Culture Collection), which carries a methylated MGMT promoter, was used as a positive control for MGMT promoter methylation. Genomic DNA extracted from peripheral blood leukocytes of normal patients was used as an unmethylated control sample. De-ionized water was used as a double-negative control. A further control reaction without any template DNA was performed together with each PCR experiment.

### Flow Cytometry Analysis of PBMCs

Patient PBMCs were isolated by apheresis as described previously (23). Briefly, 1 × 10^5^ PBMCs were collected and stained with antibodies against PD-1 (clone EH12.1 BD Biosciences, San Jose, CA, USA), CD8 (clone RPA-T8, BD Biosciences), CD3 (clone UCHT1, BD Biosciences) or their isotype antibodies, and then analyzed on a Becton Dickinson FACSCanto. The percpCy5.5-labeled CD3^+^ T cells were identified after prior gating from single and living cell according to size and granularity; then these CD3^+^ T cells were analyzed by dot-plot with CD8 and PD-1. The cells of PE-Cy7^high^ CD8^+^ and/or FITC^high^ PD-1^+^ were gated less than 1% as isotype control staining. The same gating strategy was applied in another PBMC samples to identify the populations of CD8 and PD-1 expressing T cells. All of the results were analyzed using FACSDiva software.

### Statistical Analysis

The primary end point was OS calculated from the date of the first surgery confirming GBM to the date of death. Secondary end points were progression-free survival (PFS) calculated from the date of the first surgery confirming GBM to the date of GBM recurrence confirmed by either pathology or MRI report. All statistical analyses were performed using SPSS version 20.0. Cox proportional hazards models were used to calculate hazard ratios (HRs) of recurrence or death according to the number of CD45^+^, CD4^+^, CD8^+^, and PD-1 TILs and PD-L1 of GBM. Kaplan–Meier survival analysis was used to determine the distribution of OS and PFS time, and differences were analyzed using the log-rank test.

## Results

### Patient Characteristics and Subtyping of GBM by IDH1 Mutation and MGMT Methylation

There was a total of 47 patients enrolled, of which 27 patients received ADCTA added to conventional therapy (ADCTA group) and 20 patients received conventional therapy only (reference group). Demographic and clinical data of the total patients are shown in Table 1. The mean age was 51.8 years. Detailed individual patient data of the ADCTA and reference groups including sex, age, extent of tumor resection, receiving TMZ or not, receiving radiation therapy/CCRT or not, receiving salvage gamma knife (GKS) for recurrent/residual tumor or not, Karnofsky performance score (KPS), and survival and recurrence (shown as OS and PFS in months), together with tumor expression of IDH1, MGMT, and PD-L1; PD-1^+^, CD4^+^, and CD8^+^ lymphocyte counts; and PD-1^+^/CD8^+^ ratio in lymphocytes are shown in Tables 2 and 3. Four out of the total 47 patients (6.99%) exhibited IDH1 mutation on DNA sequencing examinations, and all were R132H (CGT → CAT) mutations. There were three patients with IDH1 mutations in the ADCTA group, and one IDH1 mutation in the reference group. The O6 MGMT promoter methylation examination results showed that 7 out of 47 patients (14.9%) had unmethylated MGMT promoter, including 4 patients with unmethylated MGMT promoter in the ADCTA group and 3 patients with unmethylated MGMT promoter in the reference group. The remaining 40 patients (85.1%) exhibited methylated MGMT promoter. Therefore, in this study, clinical parameters such as IDH-1 and MGMT were well balanced between the two study groups.

**Table 1:** Demographic data of total patients and ADCTA and reference groups.

	Variable	Total (*n* = 47)		ADCTA (*n* = 27)		Reference (*n* = 20)	
				
		Number	Ratio (%)	Number	Ratio (%)	Number	Ratio (%)
Sex	Male	20	42.55	10	37.04	10	50
Female	27	57.45	17	62.96	10	50
Age (years)	≥57^a^	23	48.94	16	59.25	7	35
<57	24	51.06	11	40.75	13	65
R/T	No	12	25.53	1	3.7	11	55
	Yes	35	74.47	26	96.3	9	45
TMZ	No	13	27.66	0	0	13	65
	Yes	34	72.34	27	100	7	35
GKS	No	31	65.96	18	66.67	13	65
	Yes	16	34.04	9	33.33	7	35
CCRT	No	22	48.89	8	29.63	14	70
	Yes	23	51.11	19	70.37	6	30
ADCTA	No	20	42.55	0	0	20	100
	Yes	27	57.45	27	100	0	0
KPS	<70	18	38.3	10	37.04	8	40
	≥70	29	61.7	17	62.96	12	60
Tumor resection	Gross total	25	53.19	14	51.85	11	55
	Non-total	22	46.81	13	48.15	9	45
Isocitrate dehydrogenase 1 mutation	No	43	93.01	24	87.5	19	94.74
	Yes	4	6.99	3	12.5	1	5.26
Methylguanine-DNA methyltransferase methylation	No	7	14.89	4	14.81	3	15
	Yes	40	85.11	23	85.19	17	85

*Twenty-seven patients fitting the inclusion criteria were randomly assigned to the ADCTA group, and the remaining 20 patients were assigned to the reference group*.

*R/T, radiotherapy, completed without delay or interruption; TMZ, temozolomide; GKS, gamma knife; CCRT, concomitant chemoradiotherapy; ADCTA, autologous dendritic cell tumor antigen immunotherapy; KPS, Karnofsky Performance Score; postoperation, pre-ADCTA vaccination*.

*^a^Modified according to American Association of Neurological Surgeons: GBM occurs between the ages of 45 and 70 years*.

**Table 2:** Summary of patient data in the ADCTA group.

Lab no.	Sex	Age	WBC counts (×10^3^/µl) [3.99–10.39]	Lym counts (×10^3^/µl) [0.79–4.99]	PD-1^**+**^ [25 high-power field] lym counts	PD-1^**+**^/CD8^**+**^ ratio value	Tumor PD-L1 expression	Extent of tumor resection	Tumor location	R/T	C/T (TMZ)	GKS	CCRT	ADCTA	KPS [before]	KPS [after]	Status	OS (month) [2014/12/31]	PFS (month)	Isocitrate dehydrogenase 1 (IDH1) mutation by DNA sequencing	IDH1 IHC expression (expression%, staining intensity; DP, FP or N)	Methylguanine-DNA methyltransferase methylation
CM-02	F	47	7.95	1.32	0	0.00	No	Partial excision	Left frontal	+	+	+	+	x10	≥70	≥70	EX	67.63	4.43	Absent	0%, N	M
CM-03	F	61	6.5	1.05	14	0.21	Yes	Total excision	Right	+	+	+	+	X10	≥70	≥70	EX	30.8	16.33	Absent	0%, N	M
CM-06	M	42	5.43	1.51	0	0.00	No	Total excision	Left frontal	+	−	+	−	X10	≥70	≥70	SU	111.77	111.77	Absent	0%, N	M
CM-07	F	32	5.61	1.91	0	0.00	Yes	Total excision	Right parietal	+	+	+	+	X10	≥70	≥70	EX	66	38.97	Absent	0%, N	UM
CM-08	F	61	12.63	1.26	101	0.42	Yes	Stereotactic biopsy	Left	+	+	+	+	X10	<70	<70	EX	23.07	4.57	Absent	0%, N	UM
CM-09	M	68	10.04	1.13	126	0.29	Yes	Subtotal excision	Left	+	+	+	+	X6	<70	<70	EX	10.87	10.87	Absent	0%, N	M
CM-10	F	63	7.89	1.2	1,077	0.57	Yes	Subtotal excision	Right F-T	+	+	−	−	X10	<70	<70	EX	12.47	2.87	Absent	0%, N	M
CM-11	M	60	3.97	0.88	101	0.29	Yes	Subtotal excision	Vermis	+	+	−	+	X10	<70	<70	EX	8.2	6.8	Absent	15%, weak	M
CM-13	M	63	4.42	0.92	26	0.33	Yes	Total excision	Left F-T	+	+	+	−	X10	<70	<70	EX	17.53	14.73	Absent	0%, N	M
CM-14	M	47	3.58	0.76	7	0.01	Yes	Total excision	Left frontal	−	+	−	−	X8	<70	≥70	EX	46.13	6	Absent	0%, N	UM
CM-15	M	63	6.64	0.54	62	0.48	No	Partial excision	Right temporo-Parieto-occipital	+	+	−	−	X9	<70	<70	EX	11.47	4.2	Absent	0%, N	M
CM-17	F	64	2.74	0.54	276	0.63	Yes	Total excision	Right temporal	+	+	+	−	X7	<70	<70	EX	20.6	2.43	Absent	0%, N	M
CM-19	M	38	4.9	1.51	10	0.05	No	Stereotactic biopsy	Left temporal	−	+	−	−	X10	≥70	≥70	EX	34.33	7.7	Absent	0%, N	M
CM-20	F	46	5.65	1.82	3	0.01	No	Total excision	Left parietal	+	+	−	+	X10	≥70	≥70	SU	88.2	6.77	Present R132H	80%, strong, FP	M
CM-21	F	48	3.97	0.8	12	0.06	Yes	Total excision	Bilateral frontal	+	−	−	−	X10	≥70	≥70	EX	45.83	26.6	Absent	0%, N	M
CM-23	F	49	4.62	0.83	24	0.13	Yes	Partial excision	Right temporal	+	+	−	+	X10	≥70	≥70	EX	60.97	7.43	Absent	0%, N	M
CM-24	M	27	6.8	1.2	16	0.09	Yes	Total excision	Right frontal gyrus, bifrontal	+	+	−	+	X10	≥70	≥70	SU	66.03	66.03	Present R132H	70%, strong, FP	M
CM-25	F	58	8.22	1.54	22	0.11	No	Partial excision	Left deep parietal	+	+	−	−	X10	<70	<70	EX	31.03	31.03	Absent	0%, N	M
CM-27	F	58	4.93	1.15	153	0.14	Yes	Total excision	Right Temporal	+	−	−	−	X10	≥70	≥70	SU	64.93	64.93	Absent	0%, N	M
CM-28	M	58	6.26	2.36	36	0.08	Yes	Total excision	Left F-T-P	+	+	−	+	X10	<70	<70	EX	35.93	11.2	Absent	0%, N	M
CM-37	F	49	7.25	1.87	93	0.35	Yes	Total excision	Left parietal-occipital	−	+	−	−	X9	≥70	<70	EX	19.4	19.4	Absent	0%, N	M
CM-40	M	52	4.69	0.92	185	0.24	Yes	Excision	Right temporo-parieto-occipital	+	+	−	+	X10	≥70	≥70	EX	32.9	3.43	Absent	0%, N	M
CM-41	F	35	2.72	0.6	97	0.61	Yes	Stereotactic biopsy	Right temporal	+	+	−	+	X10	≥70	≥70	EX	32.6	6.97	Absent	0%, N	M
CM-44	F	61	6.84	0.75	343	0.61	Yes	Total excision	Right occipital	+	+	−	+	X10	≥70	≥70	EX	20.3	10.03	Absent	0%, N	M
CM-45	F	66	4.9	1.32	224	0.59	No	Total excision	Left parietal	+	+	−	+	X10	≥70	≥70	SU	20.77	2.97	Absent	0%, N	M
CM-46	M	35	10.59	1.31	0	0.00	Yes	Total excision	Right O-P	+	+	−	+	X10	≥70	≥70	EX	15.87	1.53	Present R132H	45%, N	UM
CM-47	M	31	6.68	2.22	3	0.25	Yes	Excision	Right frontal	+	+	+	+	X10	<70	N/A	EX	22.07	15.43	Absent	0%, N	M

*Symbols: +, received therapy; −, did not receive therapy; Lym, lymphocyte; R/T, radiotherapy; GKS, gamma knife; CCRT, concomitant chemoradiotherapy; ADCTA, autologous dendritic cell/tumor antigen immunotherapy; KPS, Karnofsky Performance Score (KPS before, before ADCTA; KPS after, after ADCTA); OS, overall survival; PFS, progression-free survival; tumor location: T, temporal; F, frontal; O, occipital; P, parietal; SU, patient still alive on 2014/12/31; EX, patient expired; DP, diffuse positive; FP, focal positive; N, negative; M, methylation present; UM, methylation absent*.

**Table 3:** Summary of patient data in the reference group.

Lab no.	Sex	Age	WBC counts (×10^3^/µl) [3.99–10.39]	Lym counts (×10^3^/µl) [0.79–4.99]	PD-1^**+**^ [25 high-power field] lym counts	PD-1^**+**^/CD8^**+**^ ratio value	Tumor PD-L1 expression	Extent of tumor resection	Tumor location	R/T	C/T (TMZ)	GKS	CCRT	ADCTA	KPS [before]	KPS [after]	Status	OS (month) [2014/12/31]	PFS (month)	Isocitrate dehydrogenase 1 (IDH1) mutation by DNA sequencing	IDH1 IHC expression (expression%, staining intensity; DP, FP or N)	Methylguanine-DNA methyltransferase methylation
Ref-01	M	45	12.4	1.79	7	0.09	No	Total excision	Left frontal	−	−	−	−	−	≥70	−	EX	8.53	1.63	Absent	0%, N	M
Ref-03	F	52	8.3	5.32	6	0.09	No	Stereotatic biopsy	Left basal ganglion	+	+	+	+	−	≥70	−	EX	1.6	1.6	Absent	0%, N	M
Ref-04	F	76	7.81	2.4	122	0.43	No	Total excision	Left T-P	−	−	−	−	−	≥70	−	EX	3.27	1.63	Absent	0%, N	M
Ref-05	M	54	7.37	0.81	904	0.90	No	Total excision	Left temporal	+	+	+	+	−	<70	−	EX	25.07	13.2	Absent	0%, N	M
Ref-07	F	45	10.39	1.39	40	0.39	No	Stereotatic biopsy	Left T-O	−	−	−	−	−	<70	−	EX	5.87	1.23	Absent	0%, N	M
Ref-08	M	46	9.96	1.7	41	0.08	Yes	Partial excision	Right F-P-T	+	+	+	+	−	≥70	−	EX	4.43	4.37	Absent	0%, N	M
Ref-11	M	56	8.11	1.76	48	0.62	Yes	Total excision	Gyrus	+	−	−	−	−	<70	−	EX	22.87	12.43	Absent	0%, N	M
Ref-12	M	73	10.36	1.14	34	0.16	Yes	Total excision	Right T-O	+	−	−	−	−	<70	−	EX	5.27	2.9	Absent	0%, N	M
Ref-13	M	78	9.03	1.66	170	0.56	Yes	Total excision	Left temporal	+	+	−	+	−	≥70	−	EX	9.33	2.5	Absent	0%, N	M
Ref-14	F	57	4.36	N/A	41	0.15	No	Total excision	Left frontal	+	+	−	+	−	≥70	−	EX	30.9	9.57	Absent	0%, N	UM
Ref-15	F	50	5.46	1.99	0	0.00	No	Total excision	Right frontal	−	−	−	−	−	<70	−	EX	0.2	0.2	Absent	15%, weak, N	M
Ref-17	M	25	16.46	0.92	44	0.33	Yes	Total excision	Left frontal and basal ganglion	−	−	−	−	−	≥70	−	EX	3.37	3.37	Absent	0%, N	M
Ref-20	M	49	8.79	1.16	0	0.00	Yes	Total excision	Right P-O	+	+	+	+	−	≥70	−	EX	4.63	1.7	Absent	0%, N	M
Ref-26	F	40	8.97	2.07	36	0.44	No	Total excision	Left frontal	−	−	+	−	−	≥70	−	EX	11.23	3.27	Absent	0%, N	UM
Ref-27	F	69	11.97	2.38	93	0.62	No	Excision	Right frontal	−	−	−	−	−	≥70	N/A	EX	6.4	2.9	Absent	0%, N	UM
Ref-28	F	31	16.75	1.19	27	2.45	Yes	Stereotactic biopsy	Right parietal	+	−	−	−	−	≥70	N/A	EX	8.23	8	Present R132H	100%, strong, DP	M
Ref-29	F	68	11.06	1.36	0	0.00	Yes	Stereotactic biopsy	Left parietal	−	−	+	−	−	≥70	N/A	EX	4.73	4.33	Absent	0%, N	M
Ref-30	M	78	9.58	1.69	78	0.43	No	Excision	Left frontal	−	+	+	−	−	≥70	N/A	EX	9.7	1.33	Absent	0%, N	M
Ref-31	M	52	8.14	0.83	59	0.17	No	Excision	Left parieto-temporal	−	−	−	−	−	≥70	−	EX	2.97	2.2	Absent	0%, N	M
Ref-32	F	72	8.6	2.28	16	0.16	Yes	Stereotatic biopsy	Left frontal	−	−	−	−	−	≥70	−	EX	3.47	3.47	Absent	0%, N	M

*Symbols: +, received therapy; −, did not receive therapy; Lym, lymphocyte; R/T, radiotherapy; GKS, gamma knife; CCRT, concomitant chemoradiotherapy; ADCTA, autologous dendritic cell/tumor antigen immunotherapy; KPS, Karnofsky Performance Score (KPS before, before ADCTA; KPS after, after ADCTA); OS, overall survival; PFS, progression-free survival; tumor location: T, temporal; F, frontal; O, occipital; P, parietal; SU, patient still alive on 2014/12/31; EX, patient expired; DP, diffuse positive; FP, focal positive; N, negative; M, methylation present; UM, methylation absent*.

### Expression of CD45, CD4, CD8, and PD-1 in TILs and PBMCs

**Figure 2:**
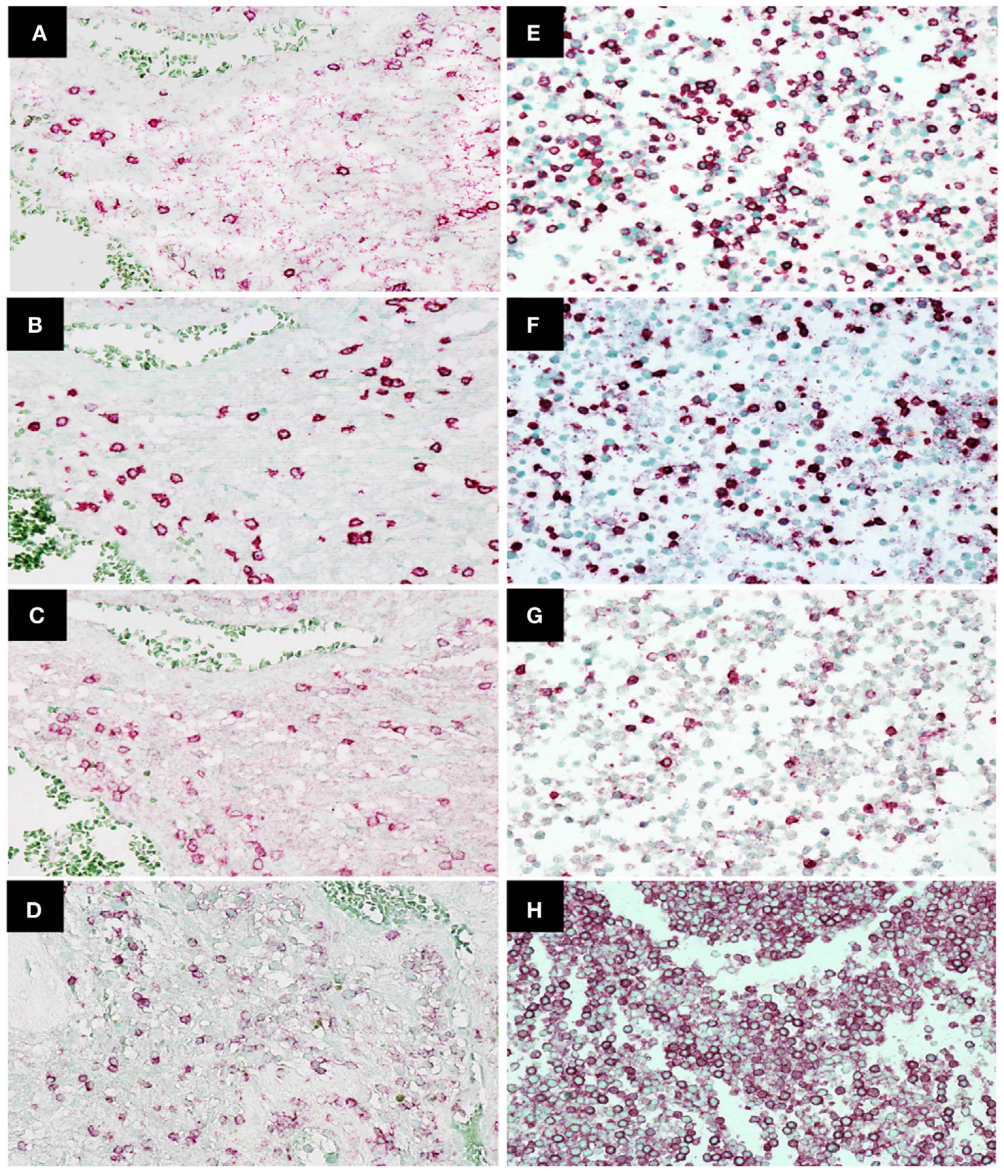
Immunohistochemistry staining of CD4, CD8, programed death 1 (PD-1), and CD45 expression in tumor-infiltrating lymphocytes in glioblastoma (GBM) tissue and peripheral blood mononuclear cells. Representative photomicrographs showing staining for CD4 **(A)**, CD8 **(B)**, PD-1 **(C)**, and CD45 **(D)** in GBM histological tissue sections (400× magnification) and CD4 **(E)**, CD8 **(F)**, PD-1 **(G)**, and CD45 **(H)** in peripheral blood mononuclear cell cytoblocks (400× magnification).

Immunohistochemistry was used to examine CD45, CD4, CD8, and PD-1 expression in TILs (47 patients) and cytoblocks of PBMCs. Morphologically identified lymphocytes (lymphoid cells exhibiting small, hyperchromatic, round to folded nucleolus with 6–9 µm nuclear diameter and thin rim of cytoplasm) positive for CD45, CD4, CD8, or PD-1 (×400) were scored based on the presence of strong circular membrane staining. Cells showing either incomplete cell membrane staining or weak staining were not scored as positive. Representative photomicrographs of TILs and PBMCs are shown in Figures 2A–H.

### Expression of PD-L1 in GBM Tumor Cells Does not Effect Prognosis

**Figure 3:**
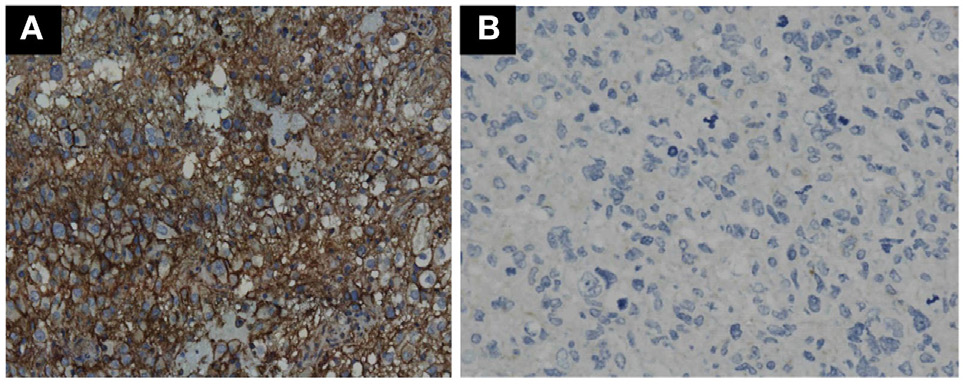
Immunohistochemistry staining pattern of positive and negative PD-L1 expression in GBM tumor cells. Representative photomicrographs showing diffuse strong cell membrane staining for PD-L1 **(A)**, and complete negative staining for PD-L1 **(B)** in GBM histological tissue sections (400× magnification).

GBM tumor cells expressing PD-L1 or not were determined by IHC. Representative photomicrographs of GBM with positive PD-L1 expression and negative PD-L1 expression are shown in Figures 3A,B. GBM tumor cells expressing PD-L1 nor not does not affect the OS and PFS of ADCTA group or reference group patients (ADCTA group OS *P* = 0.086 and PFS *P* = 0.239; reference group OS *P* = 0.376 and PFS *P* = 0.421).

### Quantitative Evaluation of CD45^+^, CD4^+^, CD8^+^, and PD-1^+^ TILs in GBM Tissue Sections and PBMC Cytoblocks

A high level of agreement was observed between the two pathologists for the counting of CD45, CD4, CD8, and PD-1 lymphocytes (κ = 0.9998, *P* < 0.001 for CD45^+^, κ = 0.9988, *P* < 0.001 for CD4^+^, κ = 0.9997, *P* < 0.001 for CD8^+^, and κ = 0.9999, *P* < 0.001 for PD-1^+^).

For all patient samples, the total count (expressed as the median and SD) of CD45^+^, CD4^+^, CD8^+^, and PD-1^+^ lymphocytes and the ratio of PD-1^+^ lymphocytes to CD8^+^ lymphocytes (PD-1^+^/CD8^+^ ratio) in both groups are shown in Table 4. The median CD45^+^, CD4^+^, CD8^+^, and PD-1^+^ lymphocyte count and median PD-1^+^/CD8^+^ ratio were used for statistical analysis. In tumor-infiltrating lymphocytes, the median CD45^+^ count was 1,782 in ADCTA group, whereas in the reference group, it was 704 per 25 HPF. The median CD4^+^ count was 262 in ADCTA group, compared to the reference group it was 130 per 25 HPF. The median CD8^+^ count was 241 in ADCTA group, and in the reference group it was 141.5 per 25 HPF. The median PD-1^+^ count was 26 in ADCTA group, whereas in the reference group, it was 40.5 per 25 HPF. The median PD-1^+^/CD8^+^ ratio in TILs in the ADCTA group was 0.21, and that in the reference group was 0.25. The median PD-1^+^/CD8^+^ ratio in cytoblocks in the ADCTA group was 0.198.

**Table 4:** The mean, median, and SD values of total count of CD45^+^, CD4^+^, CD8^+^, and PD-1^+^ lymphocytes and the ratio of PD-1^+^ lymphocytes in CD8^+^ lymphocytes (PD-1^+^/CD8^+^ ratio) for patients in this study, in ADCTA group, and reference group.

(A) Median count and SD of CD45^**+**^, CD4^**+**^, CD8^**+**^, and PD-1^**+**^ lymphocytes and the medial ratio of PD-1^**+**^ lymphocytes to CD8^**+**^ lymphocytes (PD-1^**+**^/CD8^**+**^ ratio) in tumor-infiltrating lymphocytes				

TIL	ADCTA (*n* = 27)		Reference (*n* = 20)	
		
	Median	SD	Median	SD
CD45^+^	1,782	1,318	704	1,321
CD4^+^	262	326.68	130	172
CD8^+^	241	395.95	141.5	224.1
PD-1^+^	26	213.74	40.5	196.82
PD-1^+^/CD8^+^	0.21	0.22	0.25	0.54
**(B) Median count and SD of CD4^+^, CD8^+^, and PD-1^+^ lymphocytes and the medial ratio of PD-1^+^ lymphocytes to CD8^+^ lymphocytes (PD-1^+^/CD8^+^ ratio) in PBMCs**				

**PBMC**	**ADCTA (***n*** = 18)**			
	
	**Median**		**SD**	
CD45^+^	5,184		3,278	
CD4^+^	2,966.25		1,431.55	
CD8^+^	2,980.00		1,442.72	
PD-1^+^	632.50		368.00	
PD-1^+^/CD8^+^	0.198		0.1773	

*TIL, tumor-infiltrating lymphocytes; PBMC, peripheral blood mononuclear cells; PD1, programed death 1*.

### HR Between CD45, CD4, CD8, PD-1-Presenting Lymphocytes and GBM Clinical Prognostic Features

To explore the relationship between CD45, CD4, CD8, PD-1-presenting lymphocytes and GBM clinical prognostic features, we dichotomized the CD45^+^, CD4^+^, CD8^+^, PD-1^+^ TIL counts and the PD-1^+^/CD8^+^ ratio into high and low using either the median count or the median ratio as cutoff points, as appropriate. The HR for each variable was calculated using the Cox proportion hazards model (Tables 5 and 6).

**Table 5:** Multivariate analysis using a Cox proportional hazards model for overall survival and progression free survival in ADCTA group patients.

Variable	Overall survival			Progression-free survival		
		
	*P* value	HR	95% CI	*P* value	HR	95% CI
High CD45	0.09	0.968	0.365–2.241	0.75	1.132	0.478–2.557
High CD4	0.76	1.139	0.490–2.651	0.9	1.059	0.455–2.461
High CD8	0.31	1.55	0.667–3.601	0.36	1.489	0.640–3.461
High PD-1	0.01**	3.662	1.442–9.302	0.07	2.202	0.933–5.198
High PD-1/CD8	*P* < 0.001***	11.382	3.320–35.707	0.01*	3.458	1.304–9.174
Tumor PD-L1 expression	0.1	0.354	0.103–1.219	0.248	0.528	0.178–1.563
Sex (male)	0.54	0.769	0.331–1.788	0.79	0.891	0.382–2.079
Age (≥57)	*P* < 0.001***	10.888	2.916–44.428	0.03*	2.805	1.092–7.207
With R/T	0.92	0.901	0.118–6.858	0.62	0.592	0.076–4.606
With C/T (TMZ)	0.89	1.064	0.437–2.590	0.8	0.894	0.372–2.146
With GKS	0.24	0.586	0.240–1.425	0.95	0.973	0.412–2.295
Without CCRT	*P* < 0.01**	3.799	1.516–9.516	0.04*	2.491	1.034–5.999
KPS (≥70)	0.54	0.769	0.331–1.788	0.79	0.891	0.382–2.079
Gross total tumor resection	0.07	0.452	0.193–1.058	0.02*	0.352	0.144–0.585
Presence of IHD1 mutation	0.24	0.051	0–6.964	0.291	0.036	0–17.356
Methylguanine-DNA methyltransferase methylation	0.929	0.951	0.319–2.838	0.627	0.762	0.255–2.281

*P < 0.05.

**P < 0.01.

***P < 0.001.

*HR, hazard ratio; 95% CI, 95% confidence interval; R/T, radiotherapy; TMZ, temozolomide; GKS, gamma knife; CCRT, concurrent chemoradiotherapy; ADCTA, autologous dendritic cell/tumor antigen immunotherapy; KPS, Karnofsky Performance Score; tumor location: T, temporal; F, frontal; O, occipital; P, parietal*.

**Table 6:** Multivariate analysis using a Cox proportional hazards model for overall and progression free survival in reference group patients.

Variable	Overall survival			Progression-free survival		
		
	*P* value	HR	95% CI	*P* value	HR	95% CI
High CD45	0.12	0.986	0.385–2.215	0.89	1.165	0.584–1.863
High CD4	0.46	1.415	0.569–3.520	0.91	0.951	0.379–2.383
High CD8	0.50	0.725	0.284–1.853	0.73	1.082	0.688–1.703
High PD-1	0.13	0.459	0.169–1.246	0.16	1.427	0.873–2.333
High PD-1/CD8	0.23	0.567	0.224–1.437	0.44	1.205	0.753–1.929
Tumor PD-L1 expression	0.38	0.654	0.254–1.685	0.50	1.435	0.498–4.137
Sex (male)	0.63	1.261	0.496–3.207	0.49	1.416	0.534–3.757
Age (≥57)	0.34	1.65	0.592–4.600	0.29	1.771	0.611–5.131
With R/T	0.15	0.47	0.169–1.310	0.03*	0.257	0.079–0.841
With C/T (TMZ)	0.18	0.482	0.166–1.400	0.48	0.696	0.256–1.891
With GKS	0.93	0.959	0.367–2.506	0.63	0.782	0.291–2.099
Without concomitant chemoradiotherapy	0.39	0.572	0.161–2.038	0.58	0.72	0.228–2.272
KPS (≥70)	0.73	1.185	0.460–3.049	0.47	0.692	0.253–1.893
Gross total tumor resection	0.09	0.417	0.150–1.158	0.49	0.713	0.272–1.867
Presence of IHD1 mutation	0.16	7.483	0.467–119.816	0.93	0.907	0.107–7.690
Methylguanine-DNA methyltransferase methylation	0.11	3.404	0.770–15.048	0.59	1.408	0.404–4.908

*P < 0.05.

**P < 0.01.

***P < 0.001.

A high PD-1^+^ TIL count (>median count) in the ADCTA group was associated with an increased estimated risk of death when compared with a low PD-1^+^ count (≤median count, HR = 3.662; 95% CI = 1.442–9.302; *P* = 0.01; Table 5). Moreover, a high PD-1^+^/CD8^+^ ratio (>median ratio) was associated with an increased estimated risk of death when compared to a low PD-1^+^/CD8^+^ ratio (≤median ratio) in patients in the ADCTA group (HR = 11.382; 95% CI = 3.320–35.707; *P* < 0.001, Table 5). A high PD-1^+^/CD8^+^ ratio was also associated with an increased estimated risk of disease recurrence for patients in the ADCTA group (HR = 3.458; 95% CI = 1.304–9.174; *P* = 0.01, Table 5).

Other independent prognostic factors in patients in the ADCTA group were age <57 (OS; old versus young; HR = 10.088; 95% CI = 2.916–44.428; *P* < 0.001, PFS HR = 2.805; 95% CI = 1.092–7.207; *P* = 0.03), and extent of gross tumor resection (PFS; total resection versus non-total resection; HR = 0.352; 95% CI = 0.144–0.585; *P* = 0.02). CCRT also was another prognostic factor. Patients with completion of CCRT fared better than those without CCRT in OS and PFS (without CCRT versus with CCRT; HR = 3.799; 95% CI = 1.516–9.116; *P* < 0.01 and HR = 2.491; 95% CI = 1.034–5.999; *P* = 0.04, respectively).

As for the reference group, only completion of radiotherapy without delay or interruption was an independent prognostic factor for decreased estimated risk of disease recurrence in the reference group (HR = 0.257; 95% CI = 0.079–0.841; *P* = 0.03, Table 6). None of the other factors described above were of prognostic significance in the reference group in this study.

In both groups, high CD45, high CD4 count, high CD8 count, gender, receiving radiotherapy alone, TMZ therapy alone, salvage gamma knife (GKS) treatment, or tumor cells expressing IDH1 mutation, and MGMT methylation were all not significant prognostic factors under HR evaluation.

### Prognostic Significance of CD45, CD4, CD8, and PD-1-Presenting TILs in GBM Tissue Sections

**Figure 4:**
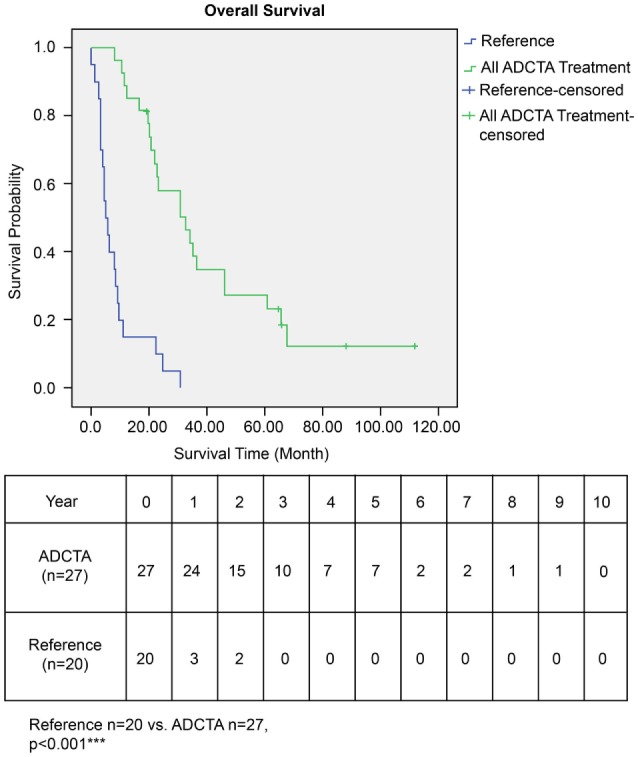
Overall survival (OS) in ADCTA versus reference group glioblastoma patients. Kaplan–Meier survival plots of the OS of 27 ADCTA patients compared to 20 reference patients.

The OS time of patients in the ADCTA group was significantly longer than that of the reference group. The median survival time in the ADCTA group was 31.0 months, whereas it was 16.0 months for the reference group (*P* < 0.001, Figure 4).

**Figure 5:**
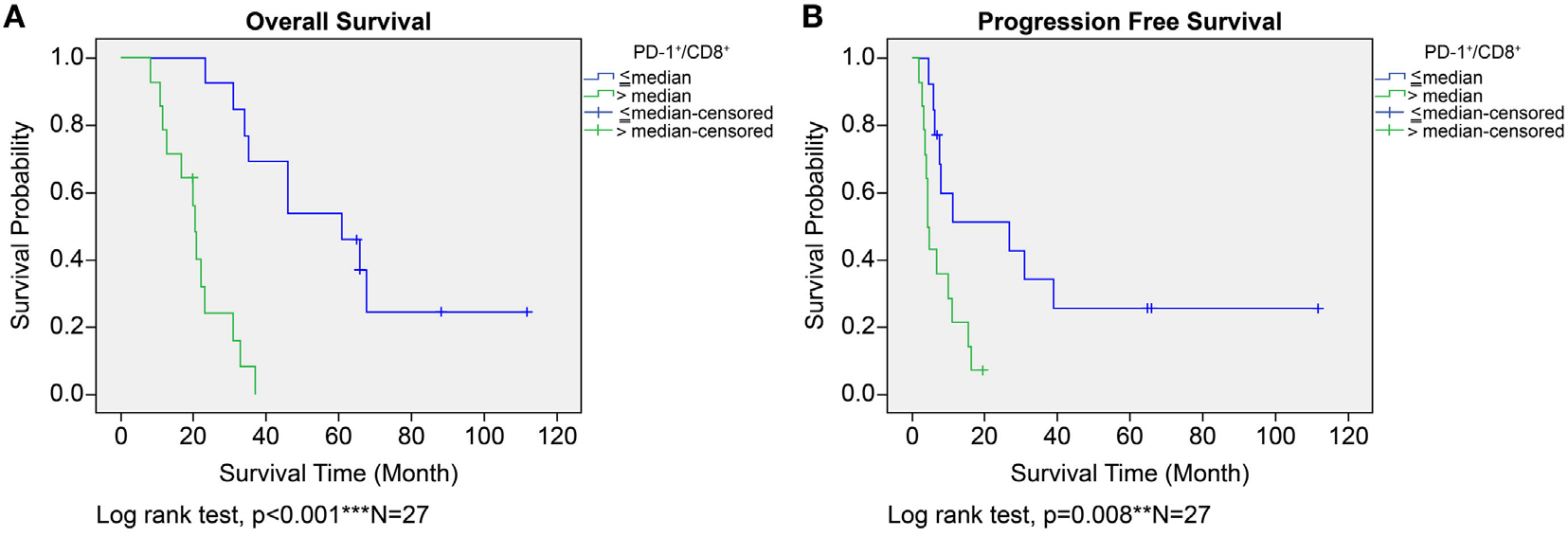
Overall survival (OS) **(A)** and progression-free survival (PFS) **(B)** by high or low PD-1^+^/CD8^+^ ratio tumor-infiltrating lymphocytes in the ADCTA group. Kaplan–Meier survival plots of OS and PFS of 14 patients with high PD-1^+^/CD8^+^ ratio compared to 13 patients with low PD-1^+^/CD8^+^ ratio patients.

**Figure 6:**
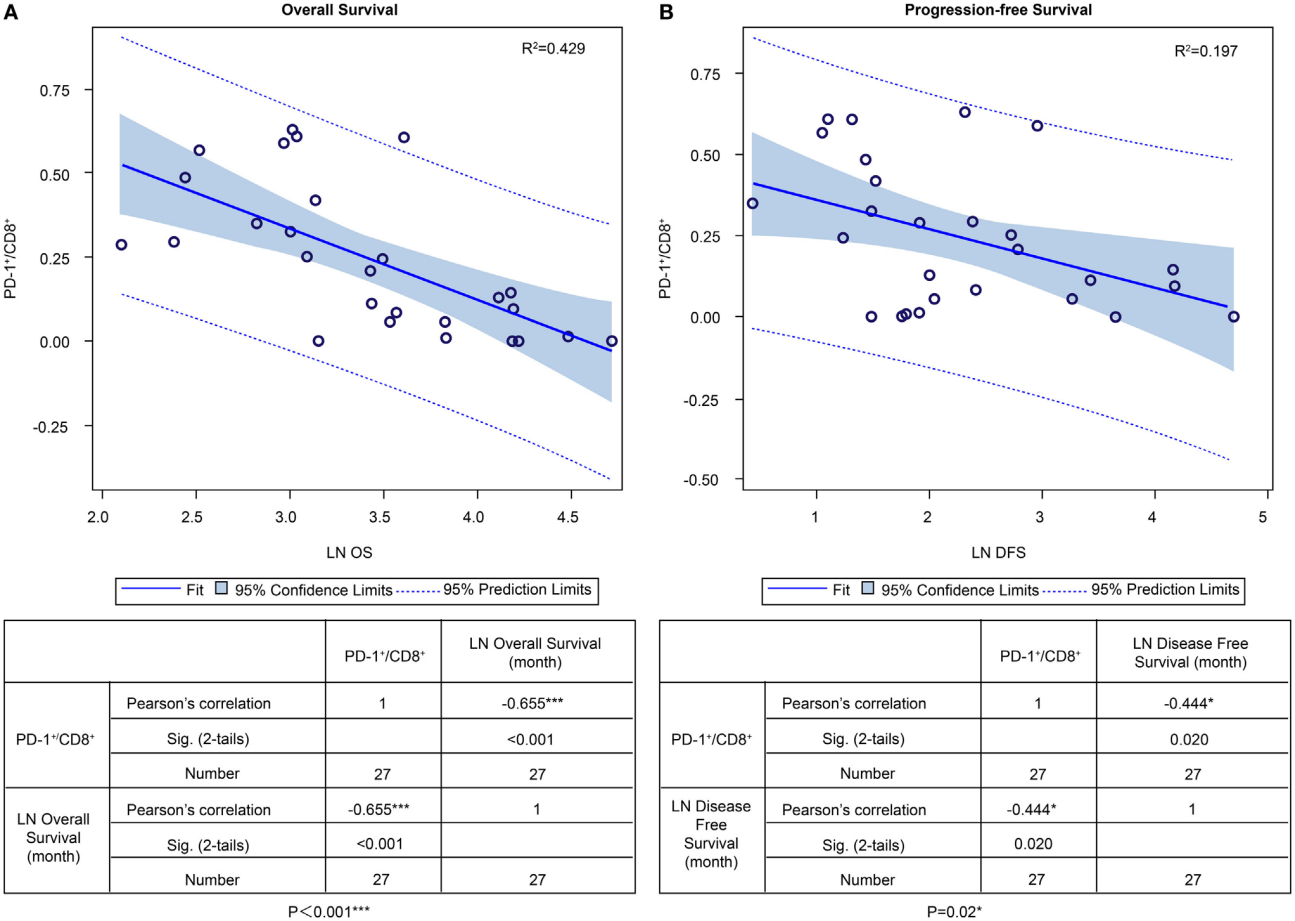
PD-1^+^/CD8^+^ ratios of individual patients’ tumor-infiltrating lymphocytes and overall survival (OS) and progression-free survival (PFS) in the ADCTA group. Pearson’s correlation of each patient’s PD1^+^/CD8^+^ ratio value and their OS **(A)** and PFS time **(B)** after natural logarithm transformation.

In the ADCTA group, in patients with a low TIL PD-1^+^/CD8^+^ ratio, the median OS was 60.97 months (95% CI = 38.7–83.24 months), whereas in patients with a high TIL PD-1^+^/CD8^+^, ratio it was 20.07 months (95% CI = 18.42–21.72 months) (Figure 5A, Kaplan–Meier estimates of OS, *P* < 0.001). As for median PFS, patients with a low TIL PD-1^+^/CD8^+^ ratio was 11.2 months (95% CI = 5.01–33.72 months), compared to patients with a high TIL PD-1^+^/CD8^+^ ratio was 4.43 months (95% CI = 3.75–5.11 months) (Figure 5B, Kaplan–Meier estimates of DFS, *P* = 0.008). Therefore, in patients with a low TIL PD-1^+^/CD8^+^ ratio, the median survival benefit was 40.9 months. The correlation between ADCTA patients’ TIL PD-1^+^/CD8^+^ ratio and survival was not just observed in dichotomized data under Kaplan–Meier survival curves. Using Pearson’s correlation coefficient, we have observed a strong negative correlation in patients’ PD-1^+^/CD8^+^ ratio and overall and DFS time after natural logarithm transformation to correct a positively skewed data distribution (Figure 6A; *r* = −0.655; *R*^2^ = 0.429; *P* < 0.001 and Figure 6B; *r* = −0.444; *R*^2^ = 0.197; *P* = 0.02). This evidence further strengthens the importance of TIL PD-1^+^/CD8^+^ ratio in GBM patients receiving ADCTA therapy.

We also examined other types of lymphocytes (CD4^−^, CD8^−^) expressing PD-1 in the ADCTA group (i.e., lymphocytes with IHC phenotype of CD4^−^/CD8^−^/PD-1^+^/CD45^+^). We counted in the slides where PD-1^+^ lymphoid cells overlapped with CD45^+^ lymphoid cells, but not with CD4^+^ or CD8^+^ lymphoid cells. The median count of these cells is 18 per 25 HPF. Lymphocytes with this immunophenotype does not affect the OS and PFS of ADCTA group patients (*P* = 0.073 and *P* = 0.249, respectively, Figure S1A in Supplementary Material).

No OS or PFS benefits were noted in patients with a low TIL PD-1^+^/CD8^+^ ratio in the reference group (*P* = 0.227 and *P* = 0.429, respectively). IHC phenotype of CD4^−^/CD8^−^/PD1^+^/CD45^+^ lymphoid cells count also does not affect the OS and PFS of reference group patients (*P* = 0.306 and *P* = 0.715, respectively, Figure S1B in Supplementary Material).

The prognostic effect of CD45^+^, CD4^+^, CD8^+^, and PD-1^+^ lymphocyte counts were not of statistical significance in both ADCTA group and reference group.

### Positive Correlation Between PBMCs, TILs, and Patient Survival in the ADCTA Group

**Figure 7:**
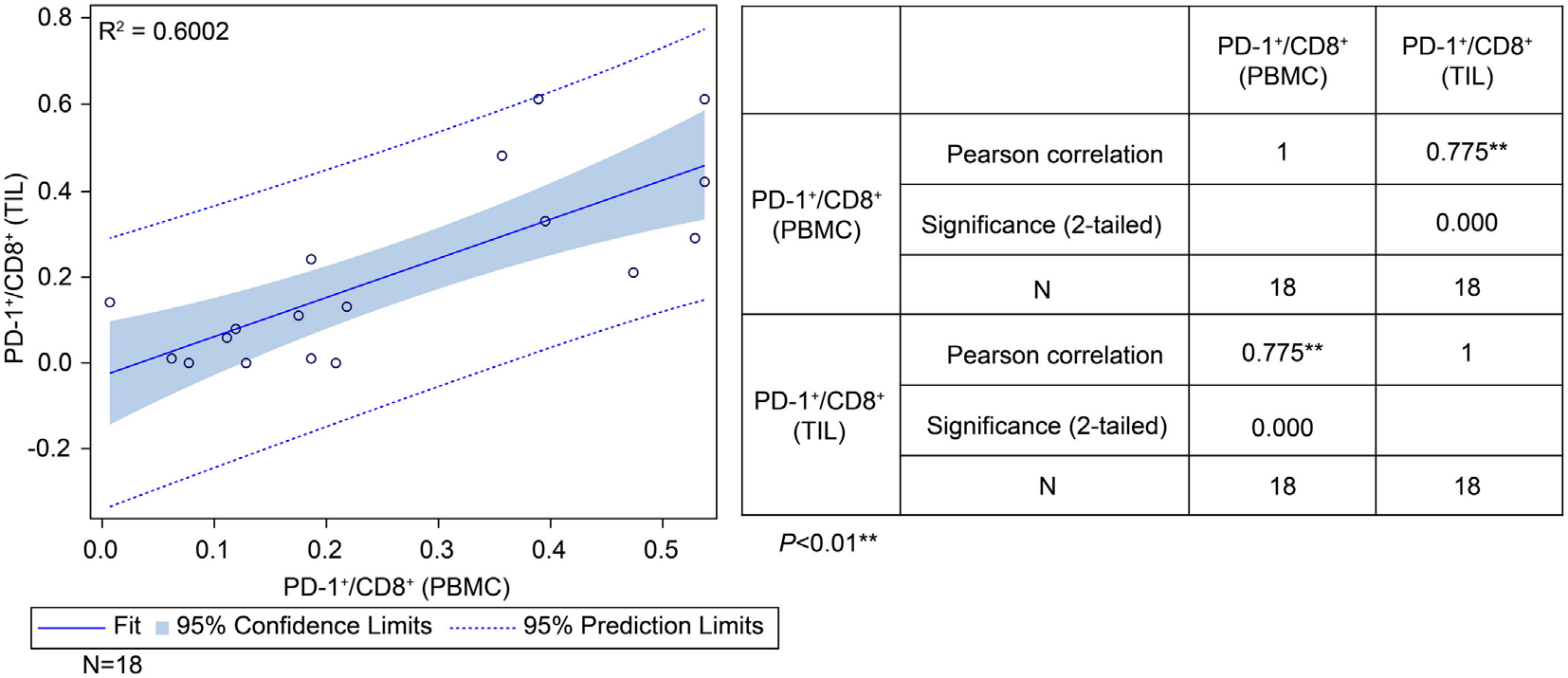
Correlation between tumor-infiltrating lymphocytes and peripheral blood mononuclear cells (PBMCs) in the ADCTA group. Pearson’s correlation between tumor-infiltrating lymphocytes and PBMCs from ADCTA patients (18 patients).

To determine the correlation between patient survival in the ADCTA group and the PD-1^+^/CD8^+^ ratio in TILs and PBMCs, we performed a chi squared test using data from the 18 (out of 27) ADCTA patients who had peripheral blood available for cytoblock preparation of PBMCs. There was a strong positive correlation between the PD-1^+^/CD8^+^ ratio found in TILs and that found in the PBMCs (*r* = 0.775; *R*^2^ = 0.6002; *P* < 0.001; Figure 7).

**Figure 8:**
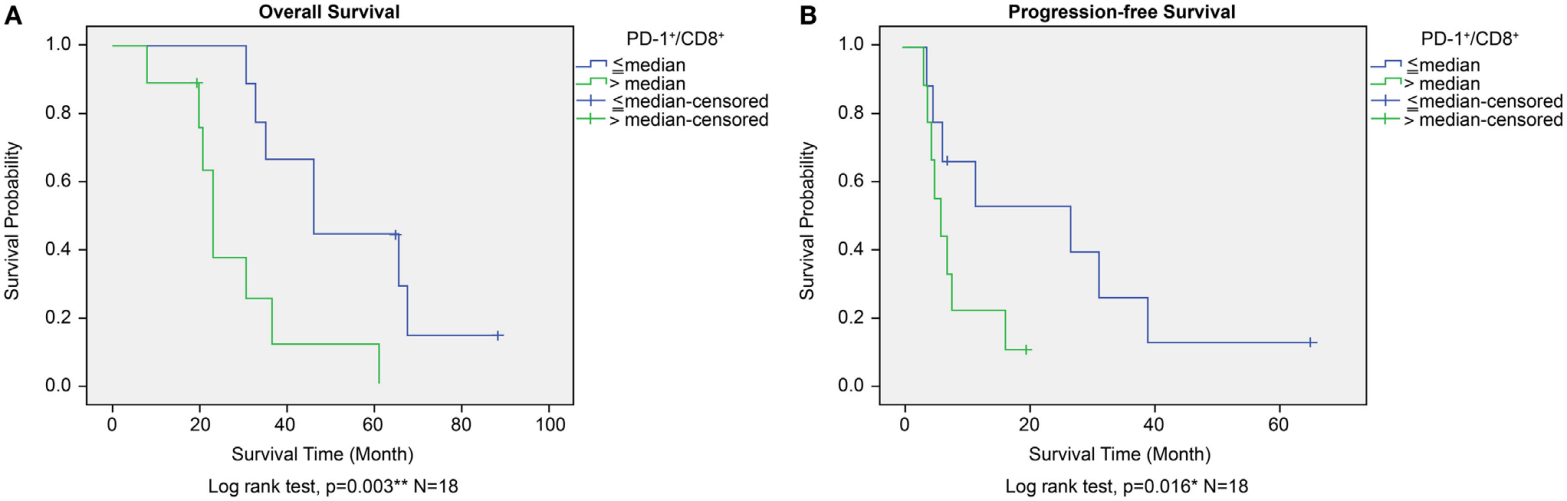
Overall survival (OS) **(A)** and progression-free survival (PFS) **(B)** according to high or low PD-1^+^/CD8^+^ ratio in peripheral blood mononuclear cells in the ADCTA group. Kaplan–Meier survival plots of OS and PFS of 10 high PD-1^+^/CD8^+^ ratio patients compared to 8 low PD-1^+^/CD8^+^ ratio patients.

The prognostic significance of CD45^+^, CD4^+^, CD8^+^, and PD-1^+^ lymphocyte counts and PD-1^+^/CD8^+^ lymphocyte ratios in PBMCs was similar to that observed for TILs from the GBM histology sections. High versus low CD45^+^, CD4^+^, CD8^+^, or PD-1^+^ lymphocyte counts in PBMCs did not correlate with any significant difference in OS or PFS. A high lymphocyte PD-1^+^/CD8^+^ ratio was associated with shorter OS as well as shortened progression free survival (PFS) (*P* = 0.003 and *P* = 0.016, respectively, Figures 8A,B). Immunophenotype CD4^−^/CD8^−^/PD-1^+^/CD45^+^ lymphocytes in PBMC also does not affect OS or PFS (*P* = 0.075 and *P* = 0.097, respectively, Figure S1C in Supplementary Material).

### Co-Expression of PD-1 Occurs in CD8^+^ Lymphocytes in TILs and PBMCs

On the basis of the significant association observed between a high PD-1^+^/CD8^+^ ratio in TILs/PBMCs and shorter patient OS or DFS in the ADCTA treatment group, we hypothesized that levels of PD-1 expression in cytotoxic CD8^+^ lymphocytes may determine the therapeutic efficacy of ADCTA therapy.

**Figure 9:**
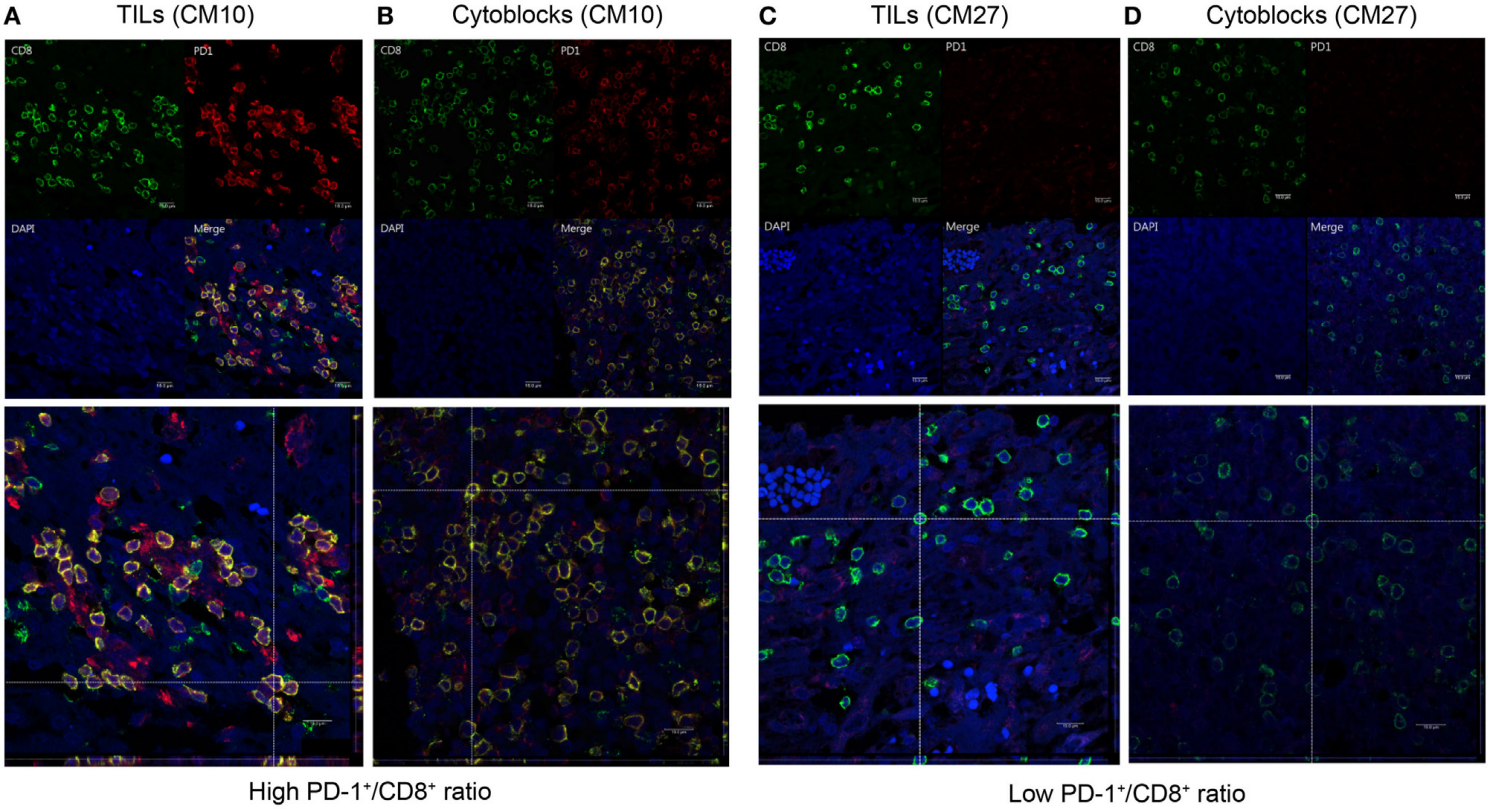
Co-expression of programed death 1 (PD-1) and CD8 in PD-1-positive and CD8-positive tumor-infiltrating lymphocytes and peripheral blood mononuclear cells (PBMCs). Paired GBM tissue sections and PBMC cytoblocks from a representative patient with a high PD-1^+^/CD8^+^ ratio were selected for immunofluorescence (IF) analysis of PD-1 and CD8 co-expression. CD8 and PD-1 expression in the TIL of GBM tissue [**(A)**, upper panel] and reconstruction of single slices of the *xz* and the *yz* planes in the *z*-axis stacked image [**(A)**, lower panel]. CD8 and PD-1 expression in PBMCs derived from the same patient [**(B)**, upper panel] and reconstruction of the same immunofluorescent slide [**(B)**, lower panel]. Paired GBM tissue sections and PBMC cytoblocks from a representative patient with a low PD-1^+^/CD8^+^ ratio were selected for IF analysis of PD-1 and CD8 co-expression. CD8 and PD-1 expression in the TIL of GBM tissue [**(C)**, upper panel] and reconstruction of single slices of the *xz* and *yz* planes in the *z*-axis stacked image [**(C)**, lower panel]. CD8 and PD-1 expression in PBMCs derived from the same patient [**(D)**, upper panel] and reconstruction of the same immunofluorescent slide [**(D)**, lower panel].

To determine whether CD8^+^ lymphocytes from patients with a high PD-1^+^/CD8^+^ ratio did indeed co-express PD-1 and CD8, we performed dual PD-1 and CD8 IF staining of GBM sections and PBMC cytoblocks followed by confocal microscopy. GBM tissue sections and PBMC cytoblocks with both high and low PD-1^+^/CD8^+^ ratios were selected for IF staining. As shown in Figure 9A, patient no. CM10 with a high PD-1^+^/CD8^+^ ratio showed abundant CD8^+^ and PD-1^+^ co-staining in lymphocytes in GBM tissue sections. Furthermore, co-localization of CD8^+^ and PD-1^+^ was observed in the merged image, confirmed by reconstruction of single slices of xz and yz planes in the z-axis stacked image (Figure 9A, left low panel). A similar phenotype was observed in the PBMC cytoblock sections from the same patient no. (CM10), in which many CD8^+^ lymphocytes co-localized with PD-1^+^ lymphocytes (Figure 9B left upper and low panels). By contrast, GBM tissue sections (Figure 9C left upper and low panels, no. CM 27) and PBMC cytoblock sections (Figure 9D upper and low panels, no. CM27) having a low PD-1^+^/CD8^+^ ratio presented abundant CD8^+^ lymphocytes but, as expected, a low number of PD-1^+^ lymphocytes, and no co-localization was observed.

### Possible Positive Association Among Frozen PBMCs’ Dual Expression PD-1^+^/CD8^+^ Cells and Their PD-1^+^/CD8^+^ Ratio in PBMC Cytoblocks, and TILs in ADCTA Group

**Figure 10:**
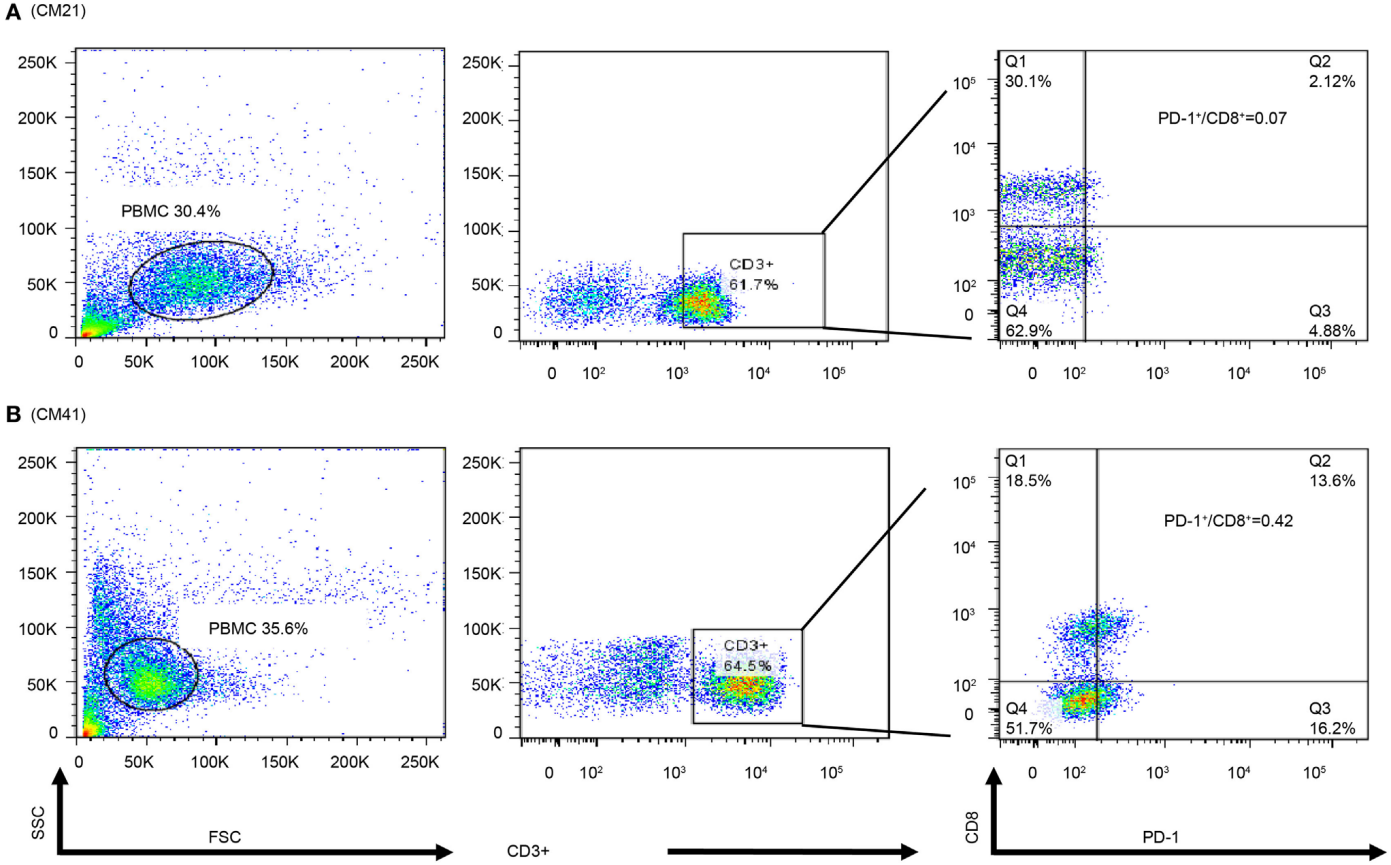
Flow cytometry of peripheral blood mononuclear cells (PBMCs) of representative patients. PBMCs (1 × 10^5^) were collected and stained with antibodies against programed death 1 (PD-1), CD8, and CD3. The percpCy5.5-labeled CD3^+^ T cells (middle panel) were gated form the size and granularity dot-plot (left panel), then these CD3^+^ T cells were analyzed by the PE-Cy7 and FITC dot-plot to identify the populations of CD8 and PD-1-expressing cells. Patient 21 had a low PD-1^+^/CD8^+^ ratio in the PBMC cytoblock **(A)**. Patient 41 was with a high PD-1^+^/CD8^+^ ratio in PBMC cytoblock **(B)**.

In order to observe the associations among frozen PBMCs, PBMC cytoblocks, and TILs, we performed flow cytometry analysis of PD-1 and CD8 in frozen PBMCs in ADCTA group from the patients with available frozen PBMCs for flow cytometry analysis. Representative results of high and low PD-1^+^/CD8^+^ ratio under flow cytometry were demonstrated. One patient (Figure 10A, CM21) had low PD-1^+^/CD8^+^ ratio (0.06 in flow cytometry and 0.07 in IHC TILs and 0.11 in cytoblocks), whereas the other patient (Figure 10B, no. CM41) had a high PD-1^+^/CD8^+^ ratio (0.41 in flow cytometry and 0.39 in cytoblocks, 0.61 in TILs). Although we could not achieve a significant statistical correlation among flow cytometry, PBMC cytoblocks, and TILs PD-1^+^/CD8^+^ ratio, there seems to be a trend in correlation between flow cytometry evaluation, PBMC cytoblock, and TILs by IHC (flow cytometry versus PBMC cytoblock *P* = 0.057 and flow cytometry versus TILs *P* = 0.068, data not shown). Our data suggest that there could be positive correlation between flow cytometry of PD-1^+^/CD8^+^ lymophocytes and PMBC cytoblocks PD-1^+^/CD8^+^ lymphocytes, and flow cytometry of PD-1^+^/CD8^+^ lymophocytes and TIL PD-1^+^/CD8^+^ lymophocytes. However, more studies are required to confirm this finding.

## Discussion

In this study of glioblastoma patients treated with adjuvant ADCTA immunotherapy compared to conventional adjuvant therapy, the treatment responsiveness in ADCTA group was found to be strongly associated with a low TIL PD-1^+^/CD8^+^ ratio within the glioblastoma tumor. Other predictors of treatment response are younger age (<57 years), gross total tumor resection, complete CCRT, and PD-1^+^ lymphocyte counts. However, CD45^+^, CD4^+^, and CD8^+^ lymphocyte count, and CD4^−^/CD8^−^/PD-1^+^/CD45^+^ immunophenotype lymphocytes does not seem to effect prognosis. Tumor expression of PD-L1 in this study also failed to predict the effectiveness of adjuvant ADCTA therapy.

From the melanoma experience (53), PD-1^+^/CD8^+^ TILs are known to express CTLA4 and Ki67 markers and lack expression of CD127, a phenotypic characteristic of exhausted T-cells. Moreover, effector cytokine production (IL2 and interferon-gamma) is also impaired PD-1^+^/CD8^+^ cells (54). In GL261 mice, DC vaccination promotes an antitumor, infiltrating T cell response but vaccination is less effective in intracranial GBM. Treatment with both DC vaccination and PD-1^+^ blockade resulted in long-term survival, whereas neither agent alone provided a survival benefit in animals with larger, established tumors (45).

To further elucidate the relationship between the PD-1/PD-L1 axis and patient prognosis, initially we also examined the expression of the PD-1 ligand PD-L1, and demonstrated that tumor PD-L1 expression was not significantly associated with prognosis in both ADCTA and reference groups. Although Nduom et al. (49) indicated that expression of PD-L1 in GBM tumor cells is associated with worse OS (49); we could not corroborate this finding in our study. The major reason could be that PD-L1 is present not only in GBM cells but also in tumor-infiltrating myeloid cells (TIMs) such as macrophages and T-regulatory cells. Some reports demonstrated that PD-L1 expression in the GBM microenvironment is contributed by TIM rather than by the tumor cells themselves. This means that patients with scarce PD-L1^+^ GBM tumor cells but with high TIM numbers surrounding the tumor environment would still have strong immune inhibition (55–57). It may also be due to the complex expression and frequent mutations of PD-L1 in glioblastoma cells (58).

Many clinical trials using DCs to treat GBM have reported an increase in CD8^+^ TILs after vaccination (59). However, in the complicated tumor microenvironment, GBM tumor cells may inhibit the immune response by the PD-1/PD-L1 pathway. The high PD-1^+^/CD8^+^ TIL ratio causes TILs to be exhausted and limits the efficacy of immunotherapy (60–63).

In this study, the median OS and PFS in the ADCTA group was 31 and 16 months without stratification of patients, respectively. But after separating the patients with a low PD-1^+^/CD8^+^ ratio, TILs extended the median OS to 61 months and PFS to 11.2 months versus high PD-1^+^/CD8^+^ ratio median OS 20.7 months and PFS 4.43 months. This drastic difference was not seen in the reference group. It could be because GBM tumor cells have strong immune system inhibition effecting immune cell proliferation and function (64). In the reference group, there are no so called “tumor antigen activated” cytotoxic T-cells to effectively kill GBM tumor cells, but in ADCTA group, there are many of these cells. So if these “tumor antigen activated” cytotoxic T-cells exhibit anergy, it will surely effect prognosis. Our results also suggest that in the PBMC of ADCTA group, although high counts of PD1+, CD4+, or CD8+ T-cells and high counts of PD-1+, but CD4^−^ and CD8^−^ lymphoid cells (including CD4 and CD8 double-negative T-cells) are identified, not all of these cells can reach the GBM tumor site and effect the ability of tumor killing by T-cells. Tumor microenvironment plays a critical role here. In order for circulating T-cells to reach the tumor microenvironment, they have to pass through the partially permissive anatomic blood–brain barrier, the desmoplastic reaction of produced by the fibroblasts surrounding the tumor cells. In addition, tumor necrosis hinders T-cell trafficking; pericytes and endothelial cells in tumor-associated angiogenesis also inhibit the circulating T-cells from reaching the tumor site.

**Figure 11:**
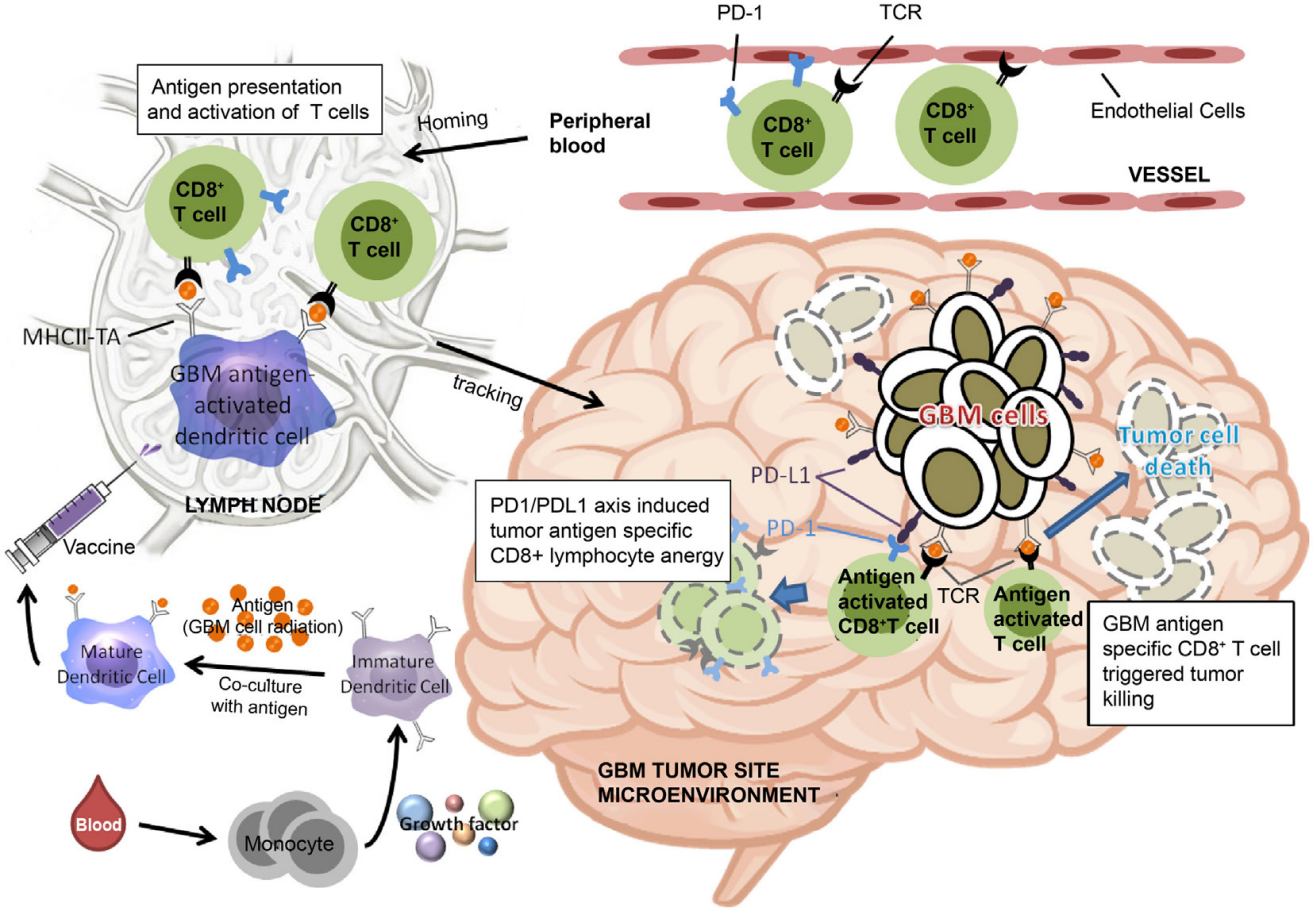
Schematic representation of dendritic cell vaccine treatment PD-1^+^ or PD-1^−^ cytotoxic T cells in the peripheral blood arrive at lymph nodes for antigen activation after vaccination. After reaching the tumor microenvironment, the efficiency of tumor cell killing by cytotoxic T cells is determined by the proportion of PD-1^+^ cytotoxic T cells.

Based on the findings of the current study, we have proposed a model to illustrate our hypothesis for predicting the effectiveness of ADCTA treatment in GBM patients. In the systemic circulation, PD-1^+^ or PD-1^−^ cytotoxic T cells in the blood will arrive at post DC vaccine injection regional lymph nodes for antigen activation. After the cytotoxic T cells reaches the tumor microenvironment, the efficiency of killing tumor cells by tumor antigen-activated cytotoxic T (CD8^+^) cells depends on the proportion of PD-1^+^ cytotoxic T (CD8^+^) cells, as shown in Figure 11. If patients have a low PD-1^+^/CD8^+^ ratio, they may have a better outcome due to reduced exhausted cytotoxic (CD8^+^) cells.

Obtaining better GBM control is critical issue. Young age, gross complete tumor resection, neuronavigation with 5 Ala, Gradel (BCNU waffle), irradiation, CCRT, IDH-1 mutation, methylated MGMT, TMZ, Avastin, and immunotherapy (2, 4, 6, 65, 66) have all been studied and may improve the tumor control. In addition, a vaccine preparation with whole tumor lysate under tumor irradiation may also improve vaccine efficacy. Furthermore, many combinatorial therapies such as immunotherapy with checkpoint blockade or with antiangiogenic therapy and cytotoxic therapy may optimize immunogenicity and target tumor adaptive immunosuppressive factors. Recent studies show an increase of the 2-year OS to 40–50%.

Programed death 1 is now a key focus in many cancer controls. In our retrospective study, the low PD-1^+^/CD8^+^ ratio may have been similar to a PD-1 blockade, resulting in less immunosuppression and improved efficacy of DC vaccination. However, PD-1 alone is insufficient for GBM control (45); DCs may be necessary to stimulate a large amount of CD8^+^ cells to attack tumor cells. Therefore, using combinatorial therapy with DCs and PD-1 blockade may provide better outcomes in patients with high immunosuppression (67, 68).

In conclusion, our study results suggest that PD-1^+^/CD8^+^ ratio is a critical factor affecting both the OS and PFS of GBM patients receiving ADCTA therapy. Other important, statistically significant factors include: age, gross total tumor removal, receiving complete CCRT, and PD-1 lymphocyte count. The TIL or PBMC PD-1^+^/CD8^+^ ratio provides a simple and feasible method of determining whether GBM patients are suitable for ADCTA adjuvant therapy. Patients who still wish to receive ADCTA immunotherapy but have a high TIL or PBMC PD-1^+^/CD8^+^ ratio may benefit from a combination therapy with anti-PD-1 and/or anti-PD-L1 monoclonal antibodies or adoptive T-cell therapy (69).

## Ethics Statement

This study was carried out in accordance with the recommendations of ethics guidelines of the institutional hospital with written informed consent from all subjects. All subjects gave written informed consent in accordance with the Declaration of Helsinki. The ethics committee at China Medical University Hospital (Taiwan) approved the study protocol (approval no. CMUH106-REC1-098).

## Author Contributions

Conception and design: C-IJ and D-YC. Development of methodology: C-IJ, S-CC, and H-JH. Acquisition of data (provided animals, acquired and managed patients, provided facilities, etc.): W-CT, and H-ML. Analysis and interpretation of data (e.g., statistical analysis, biostatistics, computational analysis): C-IJ, H-JH, W-CT, and H-ML. Writing, review, and/or revision of the manuscript: C-IJ, W-CT, S-CC, and D-YC. Administrative, technical, or material support (i.e., reporting or organizing data, constructing databases): W-CS, M-CL, and S-CC. Study supervision: C-IJ, S-CC, and D-YC.

## Conflict of Interest Statement

The authors declare that the research was conducted in the absence of any commercial or financial relationships that could be construed as a potential conflict of interest.
