# Supplementary material for: Predictors of Response to Autologous Dendritic Cell Therapy in Glioblastoma Multiforme
Source: Front Immunol. 2018 May 29;9:727. doi: 10.3389/fimmu.2018.00727 (PMC5992384; doi:10.3389/fimmu.2018.00727)
Supplement: Supplementary file 1 [file image_1.PDF]

Supplementary Material

Predictors of Response to Autologous Dendritic Cell Therapy in Glioblastoma Multiforme

Chia-Ing Jan, Mien-Chie Hung, Wan-Chen Tsai, Horng-Jyh Harn, Woei-Cherng Shyu, Ming-Chao Liu, Hsin-Man Lu, Shao-Chih Chiu*, Der-Yang Cho*

*** Correspondence:** Der-Yang Cho: d5057@mail.cmuh.org.tw; Shao-Chih Chiu: scchiu@mail.cmu.edu.tw

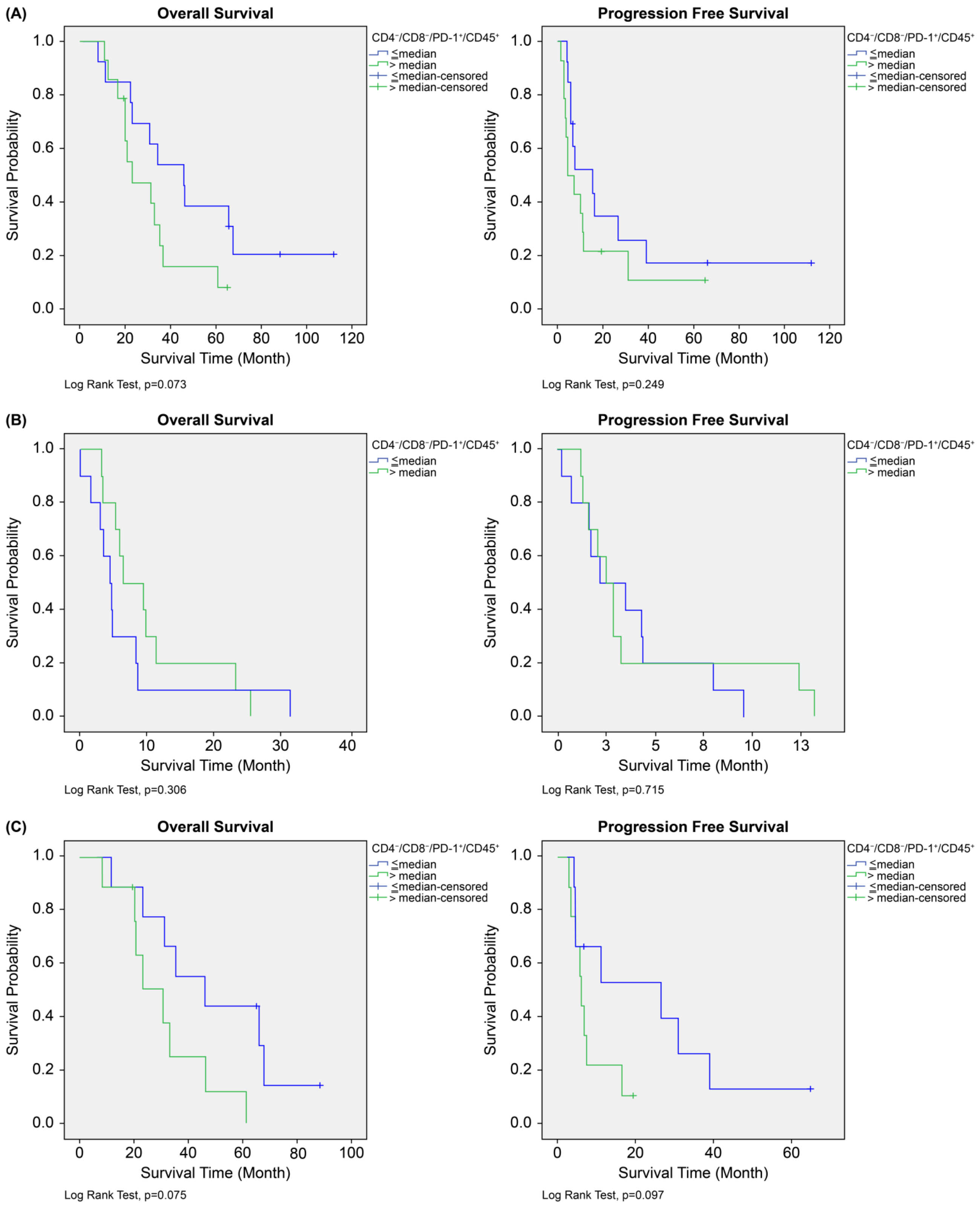

Supplementary Figure 1. (A) OS and PFS according to $CD4^{-}/CD8^{-}/PD\text{-}1^{+}/CD45^{+}$ TIL counts in the ADCTA group. Kaplan-Meier survival plots of OS and PFS in patients with high (> median) vs. low

(≤ median) CD4^{-}/CD8^{-}/PD-1^{+}/CD45^{+} lymphocyte counts (n = 14 and 13, respectively) (B) OS and PFS according to CD4^{-}/CD8^{-}/PD-1^{+}/CD45^{+} TIL counts in the reference group. Kaplan-Meier survival plots of OS and PFS in patients with high (> median) vs. low (≤ median) CD4^{-}/CD8^{-}/PD-1^{+}/CD45^{+} lymphocyte counts (n = 10 each). (C) OS and PFS by CD4^{-}/CD8^{-}/PD-1^{+}/CD45^{+} PBMC counts in the ADCTA group. Kaplan-Meier survival plots of OS and PFS in patients with high (> median) vs. low (≤ median) CD4^{-}/CD8^{-}/PD-1^{+}/CD45^{+} lymphocyte counts (N [illegible]0 and 8, respectively).
